# Rates of evolution in stress-related genes are associated with habitat preference in two *Cardamine *lineages

**DOI:** 10.1186/1471-2148-12-7

**Published:** 2012-01-18

**Authors:** Lino Ometto, Mingai Li, Luisa Bresadola, Claudio Varotto

**Affiliations:** 1 Department of Biodiversity and Molecular Ecology, IASMA Research and Innovation Centre, Fondazione Edmund Mach, Via E. Mach 1, 38010 San Michele all'Adige (TN), Italy

**Keywords:** Molecular evolution, 454 next generation sequencing, adaptive traits, habitat preference, Cardamine

## Abstract

**Background:**

Elucidating the selective and neutral forces underlying molecular evolution is fundamental to understanding the genetic basis of adaptation. Plants have evolved a suite of adaptive responses to cope with variable environmental conditions, but relatively little is known about which genes are involved in such responses. Here we studied molecular evolution on a genome-wide scale in two species of *Cardamine *with distinct habitat preferences: *C. resedifolia*, found at high altitudes, and *C. impatiens*, found at low altitudes. Our analyses focussed on genes that are involved in stress responses to two factors that differentiate the high- and low-altitude habitats, namely temperature and irradiation.

**Results:**

High-throughput sequencing was used to obtain gene sequences from *C. resedifolia *and *C. impatiens*. Using the available *A. thaliana *gene sequences and annotation, we identified nearly 3,000 triplets of putative orthologues, including genes involved in cold response, photosynthesis or in general stress responses. By comparing estimated rates of molecular substitution, codon usage, and gene expression in these species with those of *Arabidopsis*, we were able to evaluate the role of positive and relaxed selection in driving the evolution of *Cardamine *genes. Our analyses revealed a statistically significant higher rate of molecular substitution in *C. resedifolia *than in *C. impatiens*, compatible with more efficient positive selection in the former. Conversely, the genome-wide level of selective pressure is compatible with more relaxed selection in *C. impatiens*. Moreover, levels of selective pressure were heterogeneous between functional classes and between species, with cold responsive genes evolving particularly fast in *C. resedifolia*, but not in *C. impatiens*.

**Conclusions:**

Overall, our comparative genomic analyses revealed that differences in effective population size might contribute to the differences in the rate of protein evolution and in the levels of selective pressure between the *C. impatiens *and *C. resedifolia *lineages. The within-species analyses also revealed evolutionary patterns associated with habitat preference of two *Cardamine *species. We conclude that the selective pressures associated with the habitats typical of *C. resedifolia *may have caused the rapid evolution of genes involved in cold response.

## Background

Organisms adapt to different habitats through natural selection, which favors the fixation of alleles that increase the fitness of the individual that bears them. However, it is quite difficult to identify the locus/loci targeted by selection. One reason is that the number of loci involved in a particular adaptation and their phenotypic effects vary depending on the genetic architecture underlying the adaptive trait [1]. Another reason is that often the sequence and/or information about the gene(s) involved in the adaptive response is unavailable.

Two main approaches are used to identify genetic signatures of adaptive evolution and link them to phenotypic traits. The first is the *candidate gene approach*, whereby signatures of positive selection (e.g. the over-representation of specific polymorphisms) are identified using a population genetics framework in those genes previously known to be involved in the phenotypic trait of interest (e.g. [2-7]). The advantage of this approach is that the consequences of molecular variation on phenotypes can be inferred and their adaptive significance evaluated [8]. On the other hand, this approach assumes a comprehensive knowledge of the function of such genes and may neglect other genes relevant to the phenotypic trait. The second is the *genome-wide approach*, where the pattern of molecular evolution of hundreds to thousands of genes scattered across the genome are analyzed simultaneously (e.g. [9-14]). As high throughput technologies are becoming cheaper and more accessible, this approach is increasingly gaining attention from the evolutionary biology research community. An important advantage of this method is that it does not rely on prior information about gene sequence, and therefore it is well suited for studying non-model species. The possibility of performing genome scale studies in non-model organisms is indeed a powerful approach to address the genetic basis of specific adaptations, which can only be obtained by choosing the appropriate organism and ecological context. However, the typically poor knowledge about gene function in non-model organisms often prevents a comprehensive understanding of the adaptive significance of eventual signatures of positive selection. A useful compromise is to use the genome-wide approach in species closely related to well-studied model organisms, so that gene function can be inferred by comparative analyses. In this way it is possible to exploit what is known about the ecology and the life history of the species, and thus the approach is particularly suited to identifying genes involved in species-specific and habitat-specific adaptations (e.g. [11,14]).

Environmental variation is a key factor driving adaptive evolution and determining the ecological niche of a species. Plants and other sessile organisms are particularly affected by circadian and seasonal oscillations in abiotic factors. For example, sudden drops in temperature, high levels of solar irradiation and limited access to water are common sources of environmental stress, especially at high altitude in mountainous regions [15]. These stressors affect the evolution of species living in these habitats, and their capacity to adapt to these stressors ultimately determines their distribution [16-19]. To cope with drops in temperature, plants have developed a series of physiological adaptations that rely on the up- and down-regulation of cold responsive genes triggered by cold exposure (e.g. [20,21]; reviewed in [22]). Similarly, cold temperatures and high irradiation are not favorable to efficient photosynthesis, and consequently, a suite of photoprotective strategies are required for survival and reproduction at high altitudes (e.g. [23-27]). A fairly detailed understanding of the relevant regulatory pathways and gene function in the model species *Arabidopsis thaliana *now exists; however, little is known about their adaptive role, particularly in relation to the diverse habitats present along an altitudinal gradient.

In order to assess the adaptive role of cold responsive genes, as well of the genes involved in photosynthesis, we compared patterns of gene evolution in congeneric species living at different altitudes. We performed molecular evolution analyses in two closely related Brassicaceae species adapted to non-overlapping altitudinal ranges: *Cardamine resedifolia*, a perennial species usually growing under conditions of high irradiation and severe temperature oscillations between 1,500 and 3,500 meters above sea level; and *C. impatiens*, an annual nemoral species normally growing between 300 and 1,500 meters above sea level [28]. The distinct habitats associated with high and low altitudes make *C. resedifolia *and *C. impatiens *ideal species for studying the adaptive significance of the genes involved in altitude-related stress responses. It should also be noted that the two species may differ in their outcrossing rates, even though conclusive evidence is still lacking: *C. impatiens *is mainly selfing [29], although some populations have been reported as partially outcrossing [30]; *C. resedifolia *is also a predominantly selfing species, but the precise outcrossing rate is unknown [31].

The two *Cardamine *species described above are closely related to *A. thaliana *[32,33], making it possible to apply the extensive knowledge about the gene functions of this model organism to our study. Gene sequences of both *C. resedifolia *and *C. impatiens *were obtained by high-throughput sequencing technology and subsequently identified and partitioned into functional classes based on gene similarity to the *A. thaliana *orthologues. Then, we used complementary approaches, such as analyses of non-synonymous and synonymous substitutions and of their ratio, of levels of selective pressure, codon usage, and gene expression, to quantify the difference in adaptive evolution between functional classes and between species. This allowed us to infer the adaptive significance of genes involved in adaptation to cold stress and photosynthesis at high altitude and put them into an ecological context.

## Results

We obtained sequences representing the *C. resedifolia *and *C. impatiens *transcriptomes using high-throughput sequencing. Genes and their putative function were identified by comparison of the sequences obtained here to the available annotated *A. thaliana *gene sequences. To minimize the chance of mistaking a paralogue for an orthologue, we considered as triplets of putative orthologues only those consisting of reciprocal best hits, i.e. those where the three sequences were consistently found as best hit matches of one another. Eventually, we obtained 2,922 triplets of (partial) nuclear genes, with a mean (± standard error) length for the *A. thaliana *orthologues of 594.0 ± 5.8 base pairs (bp), corresponding to a mean coverage of 46.3 ± 0.5% of the full-length gene sequence. In *C. impatiens*, the mean sequence length was 592.3 ± 5.8 bp, while in *C. resedifolia *the mean sequence length was 592.2 ± 5.8 bp.

We partitioned these genes according to their putative function, and then focused our analyses on those functional classes that are associated with the adaptive response to high altitude (Additional File 1). In particular, according to the annotation in *A. thaliana*, 56 genes were involved in cold acclimation (CGO), 67 were involved in UV-B and high irradiation response (PGO), 332 were involved in general stress responses (SGO). To these three classes we added a manually compiled class that included 55 genes functionally characterized as cold responsive genes (CRG).

### Rate of molecular substitution in *Cardamine*

The analysis of the rates of nucleotide substitution revealed faster molecular evolution along the *C. resedifolia *lineage than along the *C. impatiens *lineage (Table 1). Because the rates of sequence evolution were highly correlated with the length of the *A. thaliana *orthologous gene (*P *< 0.0005 for all correlations; see Additional File 2; here and henceforth gene length is referred to the coding portion of the gene), we corrected these measures accordingly by using the residuals of such correlations (Additional File 3). While the rate of synonymous substitution, *d*_S_, was similar between lineages (Wilcoxon rank sum test, two-sided *P *= 0.1830), the rate of non-synonymous substitution, *d*_N_, was larger in the *C. resedifolia *lineage than in the *C. impatiens *lineage (*P *= 0.0001). Conversely, the ratio ω = *d*_N_/*d*_S_, was significantly larger in the *C. impatiens *than in the *C. resedifolia *lineage (*P *= 3 × 10^-9^).

**Table 1:** Rate of molecular substitution in *Cardamine*.

	*C. impatiens*		*C. resedifolia*		
			
	*n* ^a^	mean (SE)^b^	*n* ^a^	mean (SE)^b^	*P* ^c^
*d*_N_	2913	0.0073 (0.0002)	2913	0.0083 (0.0002)	0.0001
*d*_S_	2913	0.0592 (0.0007)	2913	0.0665 (0.0010)	0.1830
*d*_N_/*d*_S_	2847	0.1763 (0.0058)	2875	0.1632 (0.0044)	3 × 10^-9^

^a ^Number of genes.

^b ^Mean and standard error.

^c ^Wilcoxon test probability after correcting for gene length (partial correlation)

**Figure 1:**
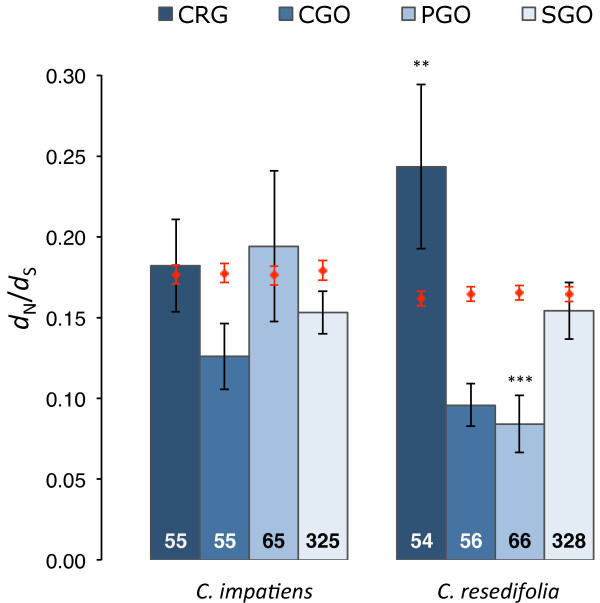
**Levels of selective pressure in *C. impatiens *and *C. resedifolia *orthologous genes**. Mean values of ω are reported for genes functionally characterized as cold responsive (CRG), and for genes annotated as involved in cold response (CGO), photosynthesis (PGO) and general stress responses (SGO). Text in bars denotes the number of genes; error bars denote the standard error of the mean. For each functional class, the mean residual values of the correlation between levels of selective pressure and *A. thaliana *gene length were compared to the mean estimated for the genes not in such functional class (identified by red dots) using a Wilcoxon test: * *P *< 0.05, ** *P *< 0.01, *** *P *< 0.001, **** *P *< 0.0001.

For each functional class, both the *d*_N _and the *d*_S _rates, as well their ratio ω, were not significantly different between the two lineages after correcting for multiple testing (corrected *P *> 0.025 for all comparisons-step-down Holm-Bonferroni method [34] applied to 8 tests for either *d*_N_, *d*_S_, and ω; Figure 1; Additional File 3). Selective constraints varied however across functional classes within the same lineage: *d*_N _was significantly lower in PGO than in other genes in the *C. resedifolia *lineage (corrected *P *= 3 × 10^-5^), and was significantly higher in CRG than in other genes in both *Cardamine *lineages (corrected *P *< 0.015 for both comparisons). The statistically significant differences were not due to random effects resulting from the small sample size of the functional classes relative to the full dataset. In fact, a bootstrap approach revealed that at most 0.2% of the randomized datasets produced Wilcoxon tests *P *values lower than those observed (10,000 replicates). Since there was a significant correlation between *d*_N _and the length of the *A. thaliana *orthologue (Spearman's ρ > -0.067, *P *< 0.0001, for both lineages), we also re-analyzed the comparisons using the residuals of the correlation. The within-lineage differences were still statistically significant, thus excluding the possibility that such differences were a result of length heterogeneity across our gene sets (Additional File 3). Similarly, the mean values of the residuals for *d*_N _and *d*_S _still did not differ between lineages, with the exception of the PGO genes, where the *d*_N _residuals were lower in *C. resedifolia *than in *C. impatiens *(corrected *P *= 0.0048).

The analysis of the levels of selective pressure, ω, provided compelling evidence that different selective forces are dominating the evolution of the four functional categories in the two *Cardamine *lineages (Figure 1; Additional File 3). Again, as a result of the correlation between substitution rates and gene length, we report the results of the residuals of such correlations. In the *C. resedifolia *lineage, the mean value of ω was significantly lower in PGO than in other genes (*P *< 0.0005), consistent with intense purifying selection. This is in sharp contrast with what observed in CRG, where the mean value of ω was significantly higher than in other genes (*P *= 0.0057), indicating either relaxed or positive selection. Instead, in the *C. impatiens *lineage, the levels of selective pressure in genes of the four functional classes were similar to those at the genome-wide level (*P *> 0.05, for all comparisons).

In *C. resedifolia*, selective pressures were different between genes involved in cold response classified as CGO and those classified as CRG. The two datasets have 12 genes in common (Additional File 1), whose mean ω did not differ from that of the remaining genes (*P *= 0.3649). However, the genes unique to CGO and CRG had a mean ω in line with what was observed in the complete datasets. That is, genes unique to the CGO category had a mean ω significantly lower than the remaining genes (*P *= 0.0056), confirming strong purifying selection in CGO. Conversely, genes exclusive to the CRG category had a mean ω significantly higher than the other genes (*P *= 0.0070), indicating either relaxed or positive selection in these genes.

The rate of evolution and the occurrence of positive selection were further investigated by analyzing the pattern of molecular substitution of each single gene. The most sensitive test for this purpose is one that employs *branch-site *models, which aim to detect positive selection affecting a few sites along particular lineages. When *C. impatiens *was used as the foreground lineage (test BS_*Ci*_) the test identified one outlier with FDR < 0.20. This number increased substantially when the *C. resedifolia *lineage was used instead (test BS_*Ci*_, FDR < 0.20), with seven genes showing evidence for positive selection along this lineage (Table 2). Two genes among these eight outliers were identified as being involved in general stress responses (not more than expected by chance; Fisher exact test, two-tailed *P *= 0.228).

**Table 2:** Fast evolving genes identified by the *branch, site*, and *branch-site *codon substitution models at a FDR threshold of 0.20.

Gene	Function	LRT^a^
AT1G07890^b^	Ascorbate peroxidase; response to salt and heat stress	BS_*Cr*_
AT1G14610	Aminoacyl-tRNA ligase	BS_*Cr*_
AT1G21680	Unknown protein	BS_*Cr*_
AT1G49750	Leucine-rich repeat family protein	S_21_, S_87_
AT1G54040^b^	Enzyme regulator; response to jasmonic acid stimulus, leaf senescence	BS_*Cr*_
AT1G71040^b^	Copper ion binding, oxidoreductase; response to phosphate starvation	B, B_*Ci*_
AT2G25840	Aminoacyl-tRNA ligase	B_*Cr*_
AT2G31610^b^	40S ribosomal protein; response to salt stress	S_21_, S_87_
AT3G06130	Metal ion binding	BS_*Cr*_
AT3G52910	Growth regulating factor, transcription activator	BS_*Cr*_
AT4G17520	Putative nuclear RNA-binding protein	S_21_, S_87_, BS_*Ci*_
AT5G06980	Unknown protein	S_21_, S_87_
AT5G20900	Jasmonate-ZIM-domain protein	BS_*Cr*_
AT5G26830	Threonyl-tRNA synthetase and ligase	B, B_*Ci*_
AT5G62680	Transport family protein	B_*Ci*_

^a ^Likelihood Ratio Tests (LRT) in which the alternative model explained the pattern of codon substitution better than the null model, and with a false discovery rate lower than 0.20. Patterns of codon substitution were tested using *branch *models (B tests), *site *models (S tests), and *branch-site *models (BS tests). B tests compared models M0 and M0'. S tests compared model M2a to M1a (S_21_) and model M8 to M7 (S_87_). BS tests were based on the *branch*-*site *model A, test 2. Subscripts in the B and BS tests indicate whether the *C. impatiens *(*Ci*) or the *C. resedifolia *(*Cr*) lineages were used as foreground branches (see main text for details).

^b ^gene of the SGO functional class.

To increase the chance of discovering interesting candidate genes, we also employed additional maximum likelihood ratio tests based on *branch *or *site *codon substitution models [35]. These tests identified several genes whose pattern was better explained by the occurrence of non-neutral evolution (Additional Files 4 and 5). *Branch *models identified three genes (with FDR < 0.20) with a pattern of nucleotide substitution that fit a model allowing two ω rates, one for the focal *C. impatiens *branch and a second for the rest of the phylogenetic tree (B_*Ci *_test). A single gene was detected if the *C. resedifolia *branch was used as the focal branch instead (B_*Cr *_test; Table 2). Using *site *models, the fit of the data under a neutral model was compared to that under a model of positive selection. In the first comparison, the neutral model admitted one class of sites with 0 < ω < 1, while the selection model allowed the presence of a second class of sites with ω > 1 (S_21 _test). The second, more powerful (less robust), approach compared more realistic models where (nearly) neutrally evolving sites were partitioned in ten classes with ω values drawn from a beta distribution (S_78 _test). The two likelihood ratio tests consistently identified four genes (with FDR < 0.20) showing evidence for the action of positive selection (Table 2).

As expected, the tests based on either the *branch, site *or *branch-site *models resulted in the detection of sets of outliers with little overlap. Among the three genes detected by more than one test, two (AT1G71040 and AT5G26830) were detected by both the B and the B_Ci _tests, and one (AT4G17520) was detected by the S_21_, S_87 _and BS_*Ci *_tests.

To exclude the possibility that substitution rate patterns were the result of orthology mis-assignment, effectively inflating interspecific divergence, we compared homologous sequences from *A. thaliana, Cardamine *and other plant species within a phylogenetic framework. Specifically, we searched for putative paralogues of the gene triplets, for each of the 15 candidate genes (Table 2). In six such cases we verified the presence of putative paralogues using BLAST searches (Additional File 6). The *A. thaliana *and the two putative *Cardamine *orthologues identified by the reciprocal best-hit approach always clustered together in the tree, supporting their orthology and the results of the likelihood ratio tests.

### Expression breadth, expression level and rate of molecular substitution

The breadth of expression of a gene, namely whether it has tissue-specific expression or is expressed broadly over more tissues, has been shown to be correlated with several patterns of molecular evolution (e.g. [36-40]).

To evaluate such effects in *Cardamine*, we correlated the levels of selective pressure, ω, to the breadth of expression measured across tissues, across developmental time points, and along stress treatments. For these analyses we used the expression patterns of *A. thaliana *orthologues as proxies for expression in *Cardamine *(see Materials and Methods), assuming that orthologous genes will maintain similar expression patterns [41-45]. These analyses found strong evidence that, in both *Cardamine *lineages, genes evolved at different rates and under different selective regimes depending on their breadth of expression (Table 3; Additional File 7). The ratio ω decreased significantly with the increase in the temporal breadth of expression, i.e. the number of flower and leaf developmental stages at which a gene was expressed (*P *< 10^-8^, for both comparisons). The same statistically significant trend held when correlating ω with the spatial breadth of expression (*P *< 10^-8^), i.e. the number of tissues/organs where the gene is expressed and the associated organ specificity index τ [46]. Because gene length was significantly correlated with both ω (ρ > 0.112, *P *< 10^-7^, for both *Cardamine *lineages) and expression breadth (ρ < -0.069, *P *< 0.0005, for all measures), we repeated our analyses using the residuals of such correlations (Additional File 7). The partial correlations coefficients were still statistically highly significant (*P *< 10^-7^, for all comparisons) and indicated that the correlations between ω and breadth of expression were independent from gene length. Thus, a broad breadth of expression imposes selective constraints limiting the possible action of positive selection. Conversely, genes expressed at one developmental time point or in specific organs evolve faster, as a result of either relaxed or positive selection.

**Table 3:** Correlation between breadth of expression and level of selective pressure, *d*_N_/*d*_S_.

Breadth type^a^	*C. impatiens*		*C. resedifolia*	
Flower development	-0.156	****^	-0.156	****^
Leaf development	-0.115	****^	-0.145	****^
Organs	-0.131	****^	-0.135	****^
Organ specificity τ	0.125	****^	0.113	****^
UV-B stress	0.061	**§	0.036	NS
Salt stress	0.057	**§	0.070	***§
Osmotic stress	0.039	*	0.057	**§
Drought stress	0.050	*	0.032	NS
Cold stress	0.043	*	0.052	**§

* Spearman's *P *< 0.05, ** *P *< 0.01, *** *P *< 0.001, **** *P *< 0.0001, NS = non significant.

^ partial correlations are significant after correcting for the length of the *A. thaliana *orthologue.

§ significant after Holm-Bonferroni multiple test correction (only for stress responses, *n *= 10)

^a ^Breadth of expression can be either spatial (i.e., number of tissues in which a gene is expressed) or temporal (when a gene is expressed, during either development or stress exposure). Note that the organ-specificity index τ is inversely correlated with the number (breadth) of organs at which a gene is expressed.

When ω was compared to the duration (i.e., the persistence) of the stress response, we obtained mixed results that depended on the stress type (Table 3). In general, the ratio ω was positively correlated to the persistence of gene expression during stress responses (Additional File 7). After a Holm-Bonferroni correction for multiple testing, ω was significantly correlated to the duration of the responses to salt and UV-B in the *C. impatiens *lineage (ρ > 0.057, corrected *P *< 0.025, for both correlations); and to the duration of the responses to salt, osmotic and cold stress in the *C. resedifolia *lineage (ρ > 0.051, corrected *P *< 0.05, for all three correlations).

In addition to the breadth of expression, the level of gene expression (in *A. thaliana*) was also an important determinant of ω (Table 4; Additional File 8). For instance, there was a statistically highly significant negative correlation between the mean gene expression and ω in both *Cardamine *lineages (ρ < 0.248, *P *< 10^-30^). This correlation also remained highly significant when the mean expression level was controlled for the effects of gene length (*P *< 10^-30^; correlation between gene length and mean expression level, ρ = -0.344, *P *< 10^-15^). Thus, we conclude that selective constraints may limit the evolution of proteins encoded by highly expressed genes in *Cardamine*.

**Table 4:** Correlation between level of selective pressure *d*_N_/*d*_S _and level of gene expression

				*C. impatiens*		*C. resedifolia*	
					
Gene FC^a^	*n* ^b^	mean (SE)^c^		ρ^**d**^	*P* ^d^	ρ^**d**^	*P* ^d^
*Mean Expression*							
All genes	2922	8.72 (0.03)	--	-0.249	****	-0.297	****
Cold response (CRG)	55	8.49 (0.21)	NS	-0.363	**	-0.310	*
Cold (CGO)	56	10.16 (0.22)	****	-0.199	NS	-0.251	NS
Photosynthesis (PGO)	67	10.29 (0.21)	****	-0.203	NS	-0.365	**
Stress (SGO)	332	9.69 (0.09)	****	-0.221	****	-0.209	***
*Maximum Expression*							
All genes	2922	11.02 (0.03)	--	-0.185	****	-0.246	****
Cold response (CRG)	55	12.51 (0.20)	****	-0.126	NS	-0.190	NS
Cold (CGO)	56	13.06 (0.24)	****	-0.184	NS	-0.097	NS
Photosynthesis (PGO)	67	12.97 (0.21)	****	-0.198	NS	-0.367	**
Stress (SGO)	332	12.26 (0.10)	****	-0.083	NS	-0.100	NS

* Spearman correlation *P *< 0.05, ** *P *< 0.01, *** *P *< 0.001, **** *P *< 0.0001, NS = non significant.

^a ^Gene functional class.

^b ^Number of genes.

^c ^Mean/maximum expression was estimated for all genes and genes included in each functional class and tested (Wilcoxon test) against mean for genes not in that functional class.

^d ^Spearman's correlation between level of gene expression and ω estimated along the two *Cardamine *lineages.

The relationship between gene expression and rate of evolution indicates that the different selective pressures we observed in genes involved in stress response may be correlated to their expression level. For instance, genes of the CGO and PGO functional classes had a calculated expression level higher than other genes (*P *< 10^-8^, for all comparisons), and also had lower ω along the *C. resedifolia *lineage than the genome-wide mean (see Figure 1). To investigate the association between gene expression and ω, we then calculated the residuals of their correlation and compared them across functional classes (Additional File 9). The corrected ω were no longer statistically significant for PGO and CGO genes, suggesting that the levels of selective pressure in these genes were highly associated with their high expression levels. Interestingly, removing the effect of expression level produced a statistically significant difference in ω between SGO genes and the other genes (*P *< 0.005, for both *Cardamine *lineages). Such differences suggests heterogeneity in the association between rates of molecular evolution and expression levels in SGO genes, likely due to the diverse nature of the stress responses to which these genes are responding. Results did not change if we analyzed the correlation between maximum, rather than mean, expression level and ω (Additional Files 8 and 9), further indicating a strong association between the expression pattern of a gene and its rate of molecular evolution.

### Codon usage in *Cardamine *genes

Synonymous codon usage can be under weak selection and lead to non-random use of the codons coding for the same aminoacid. Due to Hill-Robertson interference, such bias is expected to correlate with the occurrence of positive selection, so that it will be lower in genes that experience (recurrent) episodes of positive selection [47-49]. Consistent with this prediction, in both *Cardamine *lineages there was a statistically significant negative correlation between ω and codon bias, measured as *Fop *(frequency of the optimal codon (both ρ < -0.135, *P *< 10^-10^). This correlation also held when controlling for the effects of other determinants of codon usage, such as expression levels, gene length and GC content (e.g. as reported in [50]). For instance, in both *Cardamine *lineages, *Fop *was significantly correlated with expression level (ρ > 0.325 and *P *< 10^-50^), gene length (ρ < -0.240, *P *< 10^-39^), and GC content at the third codon position (ρ > 0.490, *P *< 10^-100^; note that most preferred codons end in either G or C, see Additional File 10). However, partial correlation coefficients (which control for dependence of these variables) between codon usage and ω remained highly significant (-0.042 < ρ < -0.121, 0.02 <*P *< 10^-10^).

**Figure 2:**
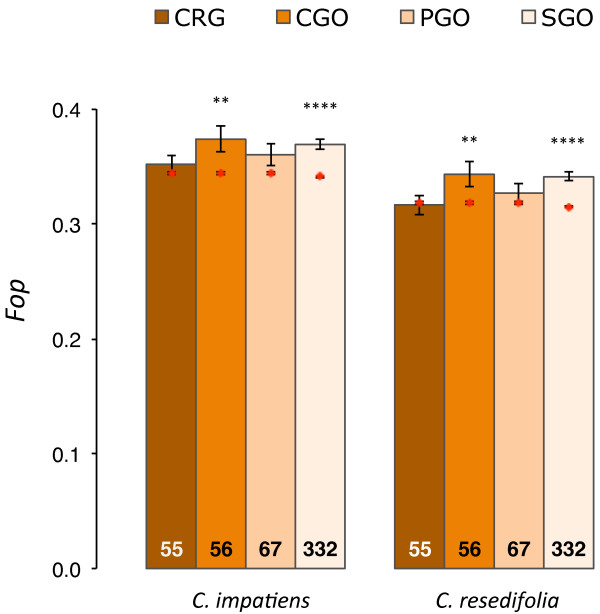
**Codon usage bias in *C. impatiens *and *C. resedifolia *orthologous genes**. Mean values of the frequency of the optimal codon (*Fop*) are reported for genes functionally characterized as cold responsive genes (CRG), and for genes annotated as involved either in cold response (CGO), photosynthesis (PGO) and general stress responses (SGO). Text in bars denotes the number of genes; error bars denote the standard error of the mean. The mean values of each functional class were compared to the mean estimated for the genes not in that functional class (identified by red dots) using a Wilcoxon test: * *P *< 0.05, ** *P *< 0.01, *** *P *< 0.001, **** *P *< 0.0001.

We then analyzed codon usage for the genes in each of the four functional classes. Genes involved in stress responses generally had larger codon bias compared to other genes (Figure 2; Additional File 11). The difference was statistically significant for CGO and SGO genes in both *Cardamine *lineages (*P *< 0.01, for both comparisons), while it was not significantly different for CRG and PGO genes (*P *> 0.05, for all comparisons). However, such differences were no longer significant when controlling for gene expression level (Additional File 11), suggesting that the expression pattern influenced codon usage more than the distinct selective pressures to which they are subject. Thus, codon usage bias does not support the frequent action of positive selection in genes of the functional classes under consideration.

## Discussion

Our analyses of protein-coding sequences highlighted lineage- and gene-specific evolutionary patterns in two species of *Cardamine *characterized by distinct habitat preferences, life-histories and, possibly, breeding systems. *C. resedifolia *and *C. impatiens *are not sister species [51]; hence, the substitution rates estimated in the present study apply to the lineages leading to the two species after the split from their common ancestor, rather than to these specific taxa as such. However, *C. resedifolia, C. impatiens*, and the clades they belong to, are characterized by divergent phenotypic and life-history traits that have profoundly shaped their evolutionary histories; for instance, the clade comprising *C. resedifolia *(group A in Figure 1 of Carlsen *et al. *[51]) is basal to the *Cardamine *radiation and includes only perennial species living in high altitude alpine habitats with well-developed petals indicating the existence of a mixed mating system. In contrast, *C. impatiens *is, to our knowledge, the only annual selfing species of its group (group C in Figure 1 of Carlsen *et al. *[51]), which includes species that are mainly found at moderate elevations. *C. impatiens *is further characterized by having very reduced or no petals, in agreement with a predominantly selfing mating system [29,30]. Thus, the observed patterns of molecular evolution reflect the differences in life history traits and habitats between the two lineages.

The effects of such differences on the rate of molecular evolution are quite complex. For instance, the rate of non-synonymous substitution was significantly higher for the *C. resedifolia *lineage than for that of *C. impatiens*. Previous studies have reported a higher substitution rate in annual plants compared to perennial plants [52-54] (but see [55]). However, our observations are in contrast with this prediction, since the annual *C. impatiens *had lower substitution rate than the perennial *C. resedifolia*. Moreover, since the rate of synonymous substitution was similar between lineages, there is no support for the hypothesis that mutation rate is higher along the *C. resedifolia *lineage than along that of *C. impatiens*.

The interspecific differences in non-synonymous substitution rates could also be the result of differences in effective population size [56,57]. For instance, low effective population size can affect the substitution rate by allowing slightly deleterious mutations to escape purifying selection and reach fixation (reviewed in [56]). Alternatively, high effective population size can increase the efficiency of positive selection by facilitating the fixation of favorable alleles in genes undergoing adaptive evolution [58]. Thus, a high dispersion in the distribution of *d*_N _across the genome is expected to be associated with a large effective population size, especially when there is recombination (e.g. [49,59]). The analysis of non-synonymous substitution fixation rate revealed that the variance in *d*_N _was significantly larger in *C. resedifolia *than in *C. impatiens *(6.6 × 10^-15 ^*vs*. 9.2 × 10^-15^; *F *test, *P *< 10^-15^, also when correcting for gene length). This difference in protein evolution heterogeneity is thus consistent with more efficient positive selection and higher effective population size in *C. resedifolia*. Both of these factors, in turn, would explain why *d*_N _is higher in this lineage than in *C. impatiens*. Preliminary analyses of recombination levels and polymorphism suggest indeed that selfing rates are lower in *C. resedifolia*, and the effective population size is slightly larger than in *C. impatiens *(Ometto and Varotto, unpublished results).

Intriguingly, mean ω was larger in *C. impatiens *than in *C. resedifolia*. The fact that in *C. impatiens *ω was considerably lower than one is compatible with either moderately positive or relaxed selection. Three lines of evidence suggest a reduced efficiency of purifying selection in *C. impatiens*, rather than pervasive positive selection at a genome-wide level. Firstly, the analyses on *d*_N _suggest that positive selection was more efficient along the *C. resedifolia *lineage than the *C. impatiens *lineage. Secondly, only one gene (using a FDR threshold of 0.20; Table 2; Additional File 5) exhibited strong evidence for site-specific positive selection along the *C. impatiens *branch. By comparison, seven genes exhibited strong evidence for site-specific positive selection along the *C. resedifolia *branch. Finally, the analysis of codon usage failed to detect differences in the efficiency of positive selection between the two lineages. However, the contrast between rates of non-synonymous substitution and of ω warrants some caution in drawing firm conclusions. The most likely explanation for the opposite patterns observed in *d*_N _and *d*_N_/*d*_S _lies on the high heterogeneity in substitution rate among genes. For instance the correlations and the coefficients of determination in the regressions between *d*_S _and ω (ρ = -0.314, *P *< 10^-15^, R^2 ^= 0.052 in *C. resedifolia*; ρ = -0.292, *P *< 10^-15^, R^2 ^= 0.037 in *C. impatiens*) and between *d*_N _and ω (ρ = 0.880, *P *< 10^-15^, R^2 ^= 0.326 in *C. resedifolia; *ρ = 0.879, *P *< 10^-15^, R^2 ^= 0.305 in *C. impatiens*), suggest that substitution rates are relatively weak predictors of the actual selective pressure experienced by a gene. The statistically significant correlation between synonymous substitution rate and ω is rather unusual: previous studies established that the correlation is caused by a combination of (i) adjacent substitutions [60] and (ii) of a bias on substitution rate estimation due to either low divergence and/or statistical methods [60,61]. Appropriate analyses will be necessary to unravel the effects of such factors in our system. The complex dynamics of substitution rates are also evident in the statistically significant correlation to gene length. The influence of gene length on sequence evolution had been previously reported for *Populus tremula *[38], stressing the importance of accounting for all diverse determinants of levels of gene and protein evolution. Additional studies, including analyses on intraspecific polymorphism, will be certainly necessary to disentangle the neutral and selective forces that have shaped such patterns [62,63].

The molecular evolution analyses in stress-related genes also revealed important lineage-specific patterns that may be associated with the distinct life-history traits and habitats of *C. resedifolia *and *C. impatiens*. For instance, similar selective regimes affected the evolution of genes involved in stress response in the *C. impatiens *lineage. Conversely, there was a correlation between the type of stress response and the rate of molecular evolution along the *C. resedifolia *lineage; for example, genes involved in photosynthesis evolved slower than other genes, consistent with selective constraints that limited the accumulation of non-synonymous substitution. This makes sense given their involvement in a process that is particularly relevant in the high altitude habitat of *C. resedifolia*. Strangely, in *C. resedifolia *selection acted in opposite directions in the two functional classes associated with cold responses. That is, compared to the genome-wide levels of selective pressure, genes that were identified as involved in cold response based on functional studies (i.e., CRG genes) displayed significantly lower levels of selective pressure, while cold responsive (CGO) genes were under more selective constraints. Since there was little overlap between the two classes (Additional File 1), the discrepancy in the levels of selective pressure between CGO and CRG genes may have captured different selective pressures acting along the cold response pathway. However, the difference may also stem from the approaches used to define the two cold-response functional classes. Specifically, the gene ontology annotations of the CGO genes were taken from the TAIR database [64], which is a less accurate source of functional information than the direct assays used to define CRG genes. For instance, the TAIR annotations may be based solely on sequence or structural similarity, and a gene may have been annotated as cold responsive because it is indirectly up- or down-regulated in plants exposed to low temperatures (e.g. more than 3,000 genes were reported as cold responsive in *A. thaliana *[20]). However, without a comprehensive knowledge on the response pathway it is difficult to evaluate their role in cold response in *Cardamine*, especially considering that their expression patterns may differ from those of *A. thaliana*.

The levels of selective pressure were also associated with the duration and pattern of gene expression. For instance, in *C. impatiens *and *C. resedifolia*, ω was lower for genes that were expressed only briefly during specific stress responses than for genes that were up-regulated over a longer period of time (as inferred by *A. thaliana *expression patterns). Most importantly, the correlation existed only for specific stress responses in each species, suggesting habitat-specific selective pressures. In particular, in *C. resedifolia*, ω was significantly affected by the duration of the responses to osmotic, salt and cold stress. The responses to these three stresses involve partially overlapping pathways [65-67], as cold stress causes membrane leakage and, as a consequence, the activation of the physiological responses also observed in high salt and osmotic stresses [68]. These results support the adaptive relevance of these genes in response to the severe temperature changes that this species experiences in alpine habitats. On the other hand, in *C. impatiens *ω was significantly correlated with the extent of up-regulation in those genes involved in UV-B stress response. *C. impatiens *is a nemoral species that is likely to be particularly sensitive to the effect of exposure to UV-B light. Therefore, it is reasonable to expect that this species has evolved response mechanisms based on gene up-regulation to cope with discontinuous UV stress. It will be extremely interesting to experimentally measure the expression of the *Cardamine *genes in the functional classes considered, as species-specific adaptive variations in expression levels cannot be excluded.

While unequal selective pressures across the stress-related functional classes were well-documented here, we detected positive selection in only a few single genes. In plants, previous studies have found evidence of genome-wide positive selection in some species (e.g. [69-71]), but in most cases there was little indication of widespread adaptive evolution (e.g. [13,72-75]). Other studies have identified positive selection of some genes involved in stress response (e.g. cold-hardiness in conifers [5]; drought stress in wild tomatoes [7]). The most likely explanation for the low rate of adaptive evolution is that most plants have low effective population size [13,76], which ultimately diminishes the efficiency of positive selection. Estimation of the effective population size of *C. resedifolia *and *C. impatiens *will be useful to verify whether this parameter can explain the scarce evidence for positive selection in our dataset.

Four methodological issues may have further resulted in an underestimation of the genes targets of positive selection. The first is that the power of codon substitution models is fairly conservative compared to that of models incorporating polymorphism data (i.e., McDonald-Kreitman test [77]). The second is that our reciprocal best-hit approach was prone to miss genes with high sequence divergence caused by adaptive evolution, as it was intended to avoid an overestimation of divergence by comparing paralogues. In particular, this approach may have overlooked duplicated genes, which are common in *A. thaliana *[78,79] and in other plants (e.g. cottonwood [80], grapevine [81]), and which can undergo sub- or neo-functionalization driven by positive selection [82]. For instance, our dataset included only ~11% of all *A. thaliana *genes, and we analyzed between 17% and 48% of the genes annotated as involved in the stress responses considered in the present study. An examination of our dataset revealed that many of the well-characterized transcription factors involved in cold response (e.g. *CBF *genes, *ICE1*) were absent. This indicates either that we are missing many of the genes upstream of the stress response pathways, or that in *Cardamine *these transcription factors may be differently regulated than in *Arabidopsis thaliana *or do not participate to the cold response. However, it is unlikely that this pathway is missing, since cold response is well-conserved across distantly related taxa (e.g. *Arabidopsis *[83]; *Citrus *[84]; *Solanum *[85]; *Poaceae *[86]). A third issue is related to the use of partial genes, which may have reduced the power of the likelihood ratio tests as a result of an insufficient number of informative sites. Finally, genes expressed at a low level, including the aforementioned transcription factors, may be missing from our dataset because of insufficient coverage or normalization. Because genes expressed at a low level are those evolving faster, this bias in gene representation could contribute to the relatively low number of rapidly evolving genes identified in this study. Deeper sequencing efforts could undoubtedly improve the situation by increasing both coverage and average transcriptome length.

One *C. impatiens *gene and seven *C. resedifolia *genes showed signatures of positive selection under the *branch-sites *model of codon substitution. The *C. impatiens *gene orthologue of AT4G17520 has not been functionally characterized in *A. thaliana*, although it is known that the protein encoded by this genes displays homology to the hyaluronan/mRNA binding protein family, a group of proteins binding both specific RNA and a high-molecular-mass polysaccharide extremely abundant in the connective tissue and extracellular matrix of animals [87]. Specific studies will be necessary to understand its role in adaptive processes in the *C. impatiens *lineage. As for the *C. resedifolia *candidate genes, no functional information is available for the orthologue of AT1G21680, which codes for a protein of unknown function with homology to TolB, a protein involved in outer membrane stability and uptake of biomolecules in *E. coli *[88]. Instead, AT3G06130 is known to code for a protein putatively involved in metal ion binding, and is similar to proteins of the heavy metal transport/detoxification superfamily. Heavy metal hyperaccumulation has been documented in several Brassicaceae species [89] and *C. resedifolia *has been reported to accumulate large quantities of nickel from the nickel-rich debris of the glacial till, where this species usually grows [90]. Therefore, it is possible that the signature of positive selection identified in this orthologue is related to high contents of heavy metals in soil experienced by *C. resedifolia*. A third gene, orthologue of AT1G14610, is a valine-tRNA synthetase. Given the multiple metabolic pathways in which valine is involved (e.g. glucosinolate [91] and pantothenate biosynthesis [92], conjugation to plant hormones [93,94]), it would be highly speculative to associate this gene to adaptive processes in the *C. resedifolia *lineage.

Based on functional evidence from *Arabidopsis *and other plant species, two other genes identified as putative targets of positive selection in *C. resedifolia *may play major roles in the response against bacterial pathogens and insect herbivory. The first gene, AT5G20900, is part of the Jasmonate-ZIM-domain protein family [95,96], whose members are involved in response to wounding and herbivory [97]. The other candidate is the orthologue of AT1G54040, which codes for the *Arabidopsis *epithiospecifier protein (ESP). ESP catalyzes the formation of simpler nitriles and epithionitriles from glucosinolates, thus modulating the release of isothiocyanate, a metabolite involved in herbivory and pathogen defense in Brassicaceae [98]. In two closely related *Boechera *species (Brassicaceae), the level of glucosinolates is negatively correlated with elevation preferences and growth rates, but positively correlated with drought tolerance [99]. However, the response to herbivory as a function of elevation is not uniform across plants, with some species experiencing more (e.g. [100]) and other less (e.g. [101-103]) damage with increase in elevation. Interestingly, a positive correlation with levels of insect herbivory was found for *C. cordifolia *exposed to full sunlight, possibly resulting from moderate water stress associated with a different insect guild compared to shadowy environments [104]. These observations provide the framework to experimentally test whether the signature of positive selection identified in AT5G20900 and AT1G54040 orthologues might be related to the higher exposition to sun, lower water availability or slower growth rate characterizing *C. resedifolia *as compared to *C. impatiens*. Interestingly, the orthologues of AT1G07890 and AT3G52910, may also be related to the light regimes characterizing *C. resedifolia *and *C. impatiens *habitats. AT1G07890 codes for a cytosolic ascorbate peroxidase (APX1) that scavenges hydrogen peroxide in plant cells [105], thus reducing the accumulation of reactive oxygen species (ROS) that cause cellular damage through protein oxidation [106]. The product of AT3G52910 is a transcriptional activator of the growth regulating factor family. Genes from this family have been demonstrated to be involved in the regulation of cell expansion and division in leaves, cotyledons and petals [107,108]. Strikingly, the product of AT3G52910 is one of the proteins oxidized in *apx1 *mutant plants in response to moderate light stress [106], indicating that it could also be part of the signaling cascade activated by ROS stress.

The *site *codon substitution models also identified putative targets of positive selection that deserve further characterization. In particular, the orthologue of AT2G31610 codes for a ribosomal protein (RPS3A) that is involved in response to salt and genotoxic stress in *A. thaliana *[109]. This gene is part of the ribosomal protein S3 family, which includes three paralogues (AT2G31610, AT3G53870 and AT5G35530) with similar sequences and function. Gene families can undergo relaxed selection immediately following duplication [110]; however, this does not seem to be the case for AT2G31610, as the phylogenetic tree clearly indicates that the duplication occurred before the split between the *Arabidopsis *and *Cardamine *lineages (Additional File 6).

Additional studies will be necessary to corroborate these findings and link the evolutionary pattern of each gene to its phenotypic effects. In particular, the use of intraspecific variation and functional analyses in the model species *A. thaliana *will be crucial to ascertaining whether positive selection or relaxed selection accelerated the evolution of these genes and their relevance in adaptive processes in *Cardamine*.

## Conclusions

Overall, our results highlight the importance of employing complementary approaches to studying the genetic bases of adaptation in non-model species. Our use of comparative genomics on congeneric species identified evolutionary patterns that aid the understanding of the extrinsic and intrinsic factors driving plant adaptation. In the case of *Cardamine*, intrinsic factors (the breeding system, demography) most likely contribute to the different levels of selective pressure in *C. resedifolia *compared to *C. impatiens *lineages. In addition, extrinsic factors (stress responses associated with habitats preferences) seem to be the primary drivers of heterogeneity in the levels of selective pressure observed among genes in *C. resedifolia*.

## Methods

### Plant material

*Cardamine resedifolia *and *C. impatiens *seeds were collected in Trentino-Alto Adige (south-eastern Alps, Italy) from the wild populations found at the localities 'Solda' (46°30'52"N, 10°33'36"E; 2660 m above mean sea level) and 'Spormaggiore' (46°13'56"N, 11°03'30"E; 420 m AMSL), respectively. After three days of cold stratification in the dark, seedlings were potted in commercial soil GS90L and grown for 15 days in a controlled environment chamber under long day photoperiod (16-h light, 25°C; 8-h dark, 23°C), with illumination of 120 μmol m^-2 ^sec^-1 ^from cool white lights.

### Sampling and RNA extraction

We enriched our mRNA library with transcripts from cold responsive genes by exposing plants to cold stress before sampling. In particular, based on the activation and kinetics of the cold responsive pathway of the close relative *Arabidopsis thaliana *(e.g. [111]), plants at the six-leaves stage were transferred to a growth chamber at 4°C with 35 μmol m^-2 ^sec^-1 ^continuous light for cold acclimation. Whole aerial parts from nine plants were harvested, pooled and plunged into liquid nitrogen immediately before, and at 15', 30', 1 h, 2 h, 3 h, 4 h, 6 h, 8 h, 12 h, 24 h, and 7 days after cold treatment (12 samples per species).

Total RNA was extracted separately from each sample using the Spectrum™ Plant Total RNA Kit (SIGMA, MO, USA), quantified in a spectrophotometer and quality controlled using electrophoretic separation in agarose gel. Approximately 1.7 μg of total RNA from each sample was pooled per species, and mRNA isolation was carried out with 20 μg of pooled RNA with Ambion Poly(A)Purist™ Kit (Life Technologies, CA, USA). mRNA quality/quantity was assessed with the RNA 6000 bioanalyzer chip (Agilent, CA, USA).

### cDNA synthesis, normalization and high throughput sequencing

Double-stranded cDNA was synthesized using the SMART cDNA library construction kit (Clontech, USA). A modified oligo-dT primer (AAGCAGTGGTATCAACGCAGAGTGGCCGAGGCGGCC(T)_20_VN*) was used for first-strand synthesis. After second strand synthesis, double-strand cDNA was purified with QIAquick PCR purification kit (Qiagen, Germany).

To enrich for rare transcripts, 2.0 μg of cDNA from each species were normalized using the Trimmer-Direct Kit (Evrogen, Russia) according to the manufacturer's instructions. 500 ng of normalized cDNA library for each species were used for 454 library preparation and simultaneously sequenced in one run on a GS FLX titanium instrument (Roche-454, USA) according to manufacturer's instructions.

The sequencing data are deposited in the EMBL ENA SRA under the accession number ERA032352.

### Reads assembly and orthologous gene set identification

The sequencing run produced 396,602 reads for the *C. impatiens *sample, with mean ± standard error (SE) length of 295.7 ± 0.2 base pairs (bp) (median = 306 bp). The run also produced 442,030 reads for the *C. resedifolia *sample, with mean length of 296.0 ± 0.2 bp (median = 306 bp). Reads were assembled using the *GS de novo assembler *software version 2.3 (Roche) using a minimum overlap of 40 bp and an identity of 100%. These stringent parameters were chosen to reduce the probability of co-aligning possible paralogous genes, and to maximize the probability of aligning reads from the same allele (although our samples consisted of inbred plants, we cannot exclude the possibility of heterozygosity in our samples). As an additional quality-control step, we only considered contigs longer than 250 bp. The assembly of the *C. resedifolia *reads resulted in 10,456 contigs with a mean length of 702.3 ± 3.6 bp, each covered at a mean depth of 29.9 ± 0.3 reads. For *C. impatiens*, the assembly resulted in 9,484 contigs with a mean length of 664.9 ± 3.7 bp, each covered at a mean depth of 29.9 ± 0.4 reads.

The identification of the orthologous sequences was done using the *C. impatiens *contigs, the *C. resedifolia *contigs and *Arabidopsis thaliana *coding sequences (TAIR9 release [64]). First, we formatted all three datasets as BLAST databases, using the dustmasker sequence filtering application for the two *Cardamine *datasets. Then we searched orthologues by running a total of six pairwise BLASTn using the BLAST+ tools suite [112]. The best-hit search was optimized using the parameters "-best_hit_overhang 0.18 -softmasking F". To reduce spurious matches, best hits were retained for the next step only if 1) at least 70% of their aligned sequences matched the respective queries; and 2) if either the query or the best hit sequences aligned for at least 60% of their length in the pairwise alignment. Finally, to reduce the chance of mistaking a paralogue for an orthologue, we identified as triplets of putative orthologues only those consisting of reciprocal best hits (RBH) [113], i.e. those where the three sequences were consistently found as best hit matches of one another. This approach resulted in a total of 2,922 triplets of putative orthologues, each triplet corresponding to a single nuclear gene. For comparison, when RBH were identified between *C. resedifolia *and *C. impatiens *only, we obtained 4,624 pairs of putative orthologues.

### Sequence alignments

After aligning the sequences of each triplet with Clustal W 2.0 [114], we extracted the portion of the alignments containing all three orthologous sequences and trimmed partial codons at the 5' and 3' ends (based on the *A. thaliana *sequence).

A potential caveat is the presence of a highly variable region present in those genes that are associated with the chloroplast. This region codes for an N-terminal transit peptide that will eventually be cleaved after targeting [115,116] and is enriched in hydroxylated residues and deficient in acidic ones [117]. Since several aminoacids can fulfill such biochemical requirements, functionally equivalent transit peptides can accumulate non-synonymous substitutions particularly fast, and may bias genome-wide and gene-specific estimates of aminoacid substitutions. Therefore, we first identified eventual transit peptides in the chloroplast-targeting *A. thaliana *genes present in our dataset using the ChloroP program [118], and subsequently removed them from the alignments.

The 2,922 *A. thaliana *aligned sequences of our final dataset had a mean ± SE length of 594.0 ± 5.8 bp (median = 531 bp; mode = 369 bp). In *C. impatiens*, the mean length was 592.3 ± 5.8 bp (median = 528 bp; mode = 384 bp). In *C. resedifolia*, the mean length was 592.2 ± 5.8 bp (median = 528 bp; mode = 384 bp).

### Gene annotation and ontology

Gene ontology (GO) and gene annotation were based on the *A. thaliana *genome annotation [119] available at TAIR (retrieved December 2009 [64]).

GO was used to discriminate genes involved in cold acclimation (CGO), in photosynthesis (as a proxy for UV-B and high irradiation response) (PGO), and those broadly involved in stress resistance (SGO; Additional File 12). In addition, we compiled a list of cold responsive genes (CRG; Additional File 13) that satisfied one of the following two conditions. The first condition was that these genes were involved in cold resistance based on known functional assays; these genes include transcription factors (*ICE1 *[120], *CBF1, CBF2 *and *CBF3 *[121-123], *ZAT12 *[21], *HOS1 *[124], and *ESK1 *[125]), and other genes active at different points along the cold acclimation response pathways (reviewed in [22]). The second condition was that the genes had to be reported as cold responsive (up-regulated in any pathway) at least twice, either in genome-wide expression studies [20,21,126-128], in their annotation description, or in the aforementioned functional studies. Such cross-validation was useful to prevent the inclusion of false-positives, since many of the ~3,000 genes identified as cold responsive in genome-wide studies are probably not primarily nor directly involved in cold resistance.

Among the 2,922 genes analyzed in the present study, 56 were included in the CGO functional class (representing 25% of all *A. thaliana *genes with such annotations), 67 in the PGO class (48%), 332 in the SGO class (17%), and 55 in the CRG class (18% of the genes included in our list). Note that some genes are present in more than one functional class, since some genes are involved in several stress-related pathways (Additional File 1). As a result of this non-independence, it was not possible to make direct statistical comparisons across functional classes.

### Analysis of the rate of molecular and protein evolution

A typical signature of positive selection is a high rate of non-synonymous substitution, *d*_N _(leading to aminoacid changes), compared to synonymous substitution, *d*_S_. This because synonymous substitutions accumulate nearly neutrally, while non-synonymous substitutions are subject to selective pressures of varying degree and sign. In general, the ratio ω = *d*_N_/*d*_S _measures the levels of selective pressure operating in a protein coding gene: the value is less than 1 if the gene is under purifying selection, equal to 1 if the gene is evolving neutrally, and greater than one if positive selection has accelerated the fixation of aminoacid changes.

For each gene, we used the program PAML 4.4 [35] to test different models of substitution rates (e.g. [129,130]). We used seven likelihood ratio tests for identifying candidates with distinct evolutionary histories, for instance genes whose substitution rates varied among lineages and/or among coding sites. For this reason, it is assumed that the outlier gene sets are, at most, only partially overlapping. For sake of completeness, we provide the results of all analyses, but we focus in particular on the *branch-site *test of positive selection, the most appropriate for detecting candidate genes under positive selection in either the *C. resedifolia *or the *C. impatiens *lineage (see below). First we compared the models that assume one or more substitution rates across the phylogeny. We estimated *d*_S _and *d*_N _between pairs of species and over all branches of the phylogenetic tree using the "one-ratio" branch model (M0), which assumes a constant *d*_N_/*d*_S _ratio, ω, across the phylogeny (with model = 0 and NSsites = 0; *A. thaliana *was used as the outgroup to *C. impatiens *and *C. resedifolia*). The likelihood of this model was compared to that of the ''free-ratio'' model (M0' [131]), which allows ω to vary among branches of the tree (model = 1 and NSsites = 0). Each comparison, i.e. twice the likelihood difference (2Δλ), was tested using a χ^2 ^test with 3 degrees of freedom (which corresponds to the number of branches minus one). In a second approach, the model M0 was compared to branch models where we assumed two ω, the first for either the *C. resedifolia *or *C. impatiens *branch, and the second for the other branches (model = 2 and NSsites = 0). In this case the likelihood ratio test was performed using 1 degree of freedom.

The occurrence of positive selection was tested by comparing the likelihoods of (nearly) neutral models to those of models that allow for the occurrence of positive selection. In a first approach we compared the likelihood of a model (M1a) that assumes two sets of sites with neutral (ω = 1) or nearly neutral evolution (0 < ω < 1), to a model (M2a) with an additional class of sites with ω > 1 (M1a: model = 0 and NSsites = 1; M2a: model = 0 and NSsites = 2). In a second more realistic approach, we compared the likelihood of a model (M7) where ten site classes have ω values drawn from a β distribution (model = 0 and NSsites = 7) to a model (M8) that incorporates an additional class of sites under positive selection (model = 0 and NSsites = 8). In this case each comparison was tested using a χ^2 ^test with 2 degrees of freedom.

Finally, we used the *branch-site *test of positive selection to detect positive selection affecting a few sites along particular lineages (i.e. in the foreground branches). In this test (branch-site model A, test 2 [132]), ω can vary both among sites in the protein and across branches on the tree (model = 2 NSsites = 2). We set as foreground branches either the *C. resedifolia *or the *C. impatiens *lineages and allowed two ω along the branches. The null model fixes ω_2 _to one (fix_omega = 1, omega = 1), while the positive selection model allows ω_2 _to be larger than one (fix_omega = 0, omega = 1). The likelihood ratio test had one degree of freedom.

To account for multiple testing, for each likelihood ratio test, we estimated the false discovery rate (FDR) using the qvalue package [133] implemented in R [134]. Only genes with a FDR lower than 0.20 were discussed further in the main text. This threshold was chosen to allow between-tests comparisons and to account for the different power of the likelihood ratio tests (e.g. the S_21 _test is less powerful than the S_87 _test). Moreover, the fact that we used only partial genes in our analyses, and that the phylogeny included only three species, may have considerably reduced the power of these tests (see PAML documentation [35]).

Rates of synonymous and non-synonymous substitution used in the analyses were those estimated by the ''free-ratio'' model along the *C. resedifolia *and the *C. impatiens *branches.

### Breadth and levels of expression

Since gene expression data are not yet available for *C. impatiens *and *C. resedifolia*, here we assumed that data for the closely related species *A. thaliana *can serve as a proxy for expression levels in all three species. This is reasonable, given that a recent study suggests quite similar patterns of expression between *A. thaliana *and the far more divergent species *Silene latifolia *[135].

For most of the genes in our list we could calculate the breadth of expression based on their expression levels collected across the developmental gradient in several organs of *A. thaliana *[136]. The expression levels were originally inferred from the intensity of each gene's hybridization onto an Affymetrix microarray [136]. For our analyses, log2 transformed gene expression intensities (which were) were back-transformed by calculating the power of two of their values. The spatial breadth of expression was estimated as the number of organs (minimum 1, maximum 14) where the gene expression value was larger than 75 (arbitrarily chosen as threshold to reduce false positives, see [40]; values ranged from 0 to 66,760, with a median of 460). For organs represented by more developmental stages, we considered a combined value that equaled 1 only when the gene was expressed in at least one developmental stage. In a second approach, we used the organ specificity index τ [46], which also includes the information on the level of expression. This index has a value of 0 when the gene is expressed equally across organs, while it approaches 1 when the gene has organ-specific expression. We also estimated the temporal breadth of expression in leaves and flowers as the number of developmental time points in which the gene was expressed at a level larger than 75.

In addition, we calculated the breadth of expression as a function of the kinetics of a gene in the presence of an abiotic stress. Our rationale for choosing this estimate is that genes expressed briefly during a stress response may be subject to different selective pressures compared to those expressed continuously. For our purpose, we used the AtGenExpress dataset [128], which contains time series of expression data collected from plants subjected to one of the following stress treatments: UV-B radiations, drought, salt, cold and osmosis. For every stress treatment, we assigned to each gene a value corresponding to the number of time-points at which its expression in treated plants was at least 3 times higher than in control plants [128].

Finally, we estimated the mean and maximum levels of expression of the genes from data reported in [136]. Because stress responses are typically not constitutive, using maximum expression levels reduces the possibility to overlook transient peaks of expression.

### Codon usage analysis

We estimated codon bias using the frequency of optimal codons (*Fop *[137], where stronger synonymous codon usage bias is identified by larger *Fop *values. This index was calculated using the program CodonW (version 1.4.2 [138]). First we inferred the preferred codon usage for each species using the correspondence analysis of relative synonymous codon usage approach as implemented in CodonW. Briefly, putative optimal (preferred) codons are identified as those that are significantly overrepresented in genes with high codon bias compared to those with low bias [139]. To avoid a bias due to the heterogeneous composition of our gene dataset (enriched in stress related genes, see above), we inferred the preferred codons only from genes annotated as ribosomal (*n *= 77), which, being highly expressed, should be enriched in optimal codons. Similarly, we discarded 11 (partial) genes shorter than 100 codons [140,141]. We then let CodonW to automatically identify codon usage on the 50% highest- and lowest-biased genes (see Additional File 10 for a list of putative optimal codons in *Cardamine*). This percentage was chosen because in *A. thaliana *it maximized the agreement between our estimate and what is reported in the literature [139]. Indeed, optimal codons identified by using higher or lower percentages of the ribosomal genes, or the 5% highest- and lowest-biased genes among all those sequenced, had a lower agreement (data not shown). Therefore, we can assume that the preferred codon usage patterns identified for the two *Cardamine *species are also very close to the real ones.

## Authors' contributions

LO contributed to develop the design of the study, performed the bioinformatics and statistical analyses, and drafted the manuscript. ML carried out all molecular work related to the 454 sequencing, and participated in manuscript drafting. LB contributed to the sequence evolution analyses. CV conceived and coordinated the study, participated in data analysis, and drafted the manuscript. All authors read and approved the final manuscript.

## Supplementary Material

Additional file 1 **Genes in functional classes**. Venn-diagrams showing the numbers of genes in the functional classes considered in this study. Click here for file

Additional file 2 **Correlation between substitution rate and gene length**. Plot showing the correlation between the length of the *A. thaliana *orthogous gene and the substitution rate in *C. impatiens *and *C. resedifolia *genes. Click here for file

Additional file 3 **Rate of synonymous and non-synonymous substitution in *Cardamine *genes. I**. Mean substitution rate in *C. resedifolia *and *C. impatiens *genes included in the four functional classes considered in this study. Statistical comparisons within and between lineages are also reported, including those calculated for values corrected for gene length. Click here for file

Additional file 4 **Number of genes identified by likelihood ratio tests that compared *branch, site *and *branch*-*site *codon substitution models**. Number of genes identified (at decreasing probabilities thresholds) as putative targets of positive selection by likelihood ratio tests based on *branch *models (B tests, Table S4.1), *site *models (S tests, Table S4.2), and *branch-site *models (BS tests, Table S4.3). Click here for file

Additional file 5 **Description of the top ten genes identified by likelihood ratio tests that compared various codon substitution models**. Top ten genes identified as putative targets of positive selection by likelihood ratio tests based on *branch *models (B tests, Tables S5.1-S5.3), *site *models (S tests, Tables S5.4-S5.5), and *branch-site *models (BS tests, Tables S5.6-S5.7). Click here for file

Additional file 6 **Phylogenetic trees of candidate genes**. Phylogenetic trees for the five genes that were putative targets for positive selection (at FDR < 0.20) according to likelihood ratio tests based on the *site *and *branch-site *codon substitution models implemented in PAML. Click here for file

Additional file 7 **Correlation between the temporal breadth of expression and levels of selection**. Spearman's correlations between levels of selection and both the spatial and temporal breadth of expression. Click here for file

Additional file 8 **Correlation between rate of molecular evolution and level of gene expression**. Mean and maximum expression levels, and Spearman's correlations between such expression levels and levels of selection for the genes included in the four functional classes considered in this study. Click here for file

Additional file 9 **Rate of synonymous and non-synonymous substitution in *Cardamine *genes. II**. Mean substitution rate in *C. resedifolia *and *C. impatiens *genes included in the four functional classes considered in this study. Comparisons of the values corrected for expression levels. Click here for file

Additional file 10 **Optimal codons in *Cardamine***. Putative optimal codons identified in *C. resedifolia *and *C. impatiens*. Click here for file

Additional file 11 **Codon usage bias in *Cardamine *genes**. Mean codon usage bias, measured as *Fop*, in the four functional classes considered in this study. Statistical results of the comparisons between functional classes and species are also reported. Click here for file

Additional file 12 **Definition of the Gene Ontology (GO) terms associated with the functional classes analyzed in this study**. GO terms and associated function used to identify genes in our dataset that were putatively involved in cold response, photosynthesis, and general stress response. Click here for file

Additional file 13 **Cold responsive genes**. List of genes identified as involved in cold response in previous studies. Click here for file
