# Supplementary material for: Rates of evolution in stress-related genes are associated with habitat preference in two Cardamine lineages
Source: BMC Evol Biol. 2012 Jan 18;12:7. doi: 10.1186/1471-2148-12-7 (PMC3398273; doi:10.1186/1471-2148-12-7)
Supplement: Additional file 1 — Genes in functional classes. Venn-diagrams showing the numbers of genes in the functional classes considered in this study. [file 1471-2148-12-7-S1.PDF]

Additional File 1

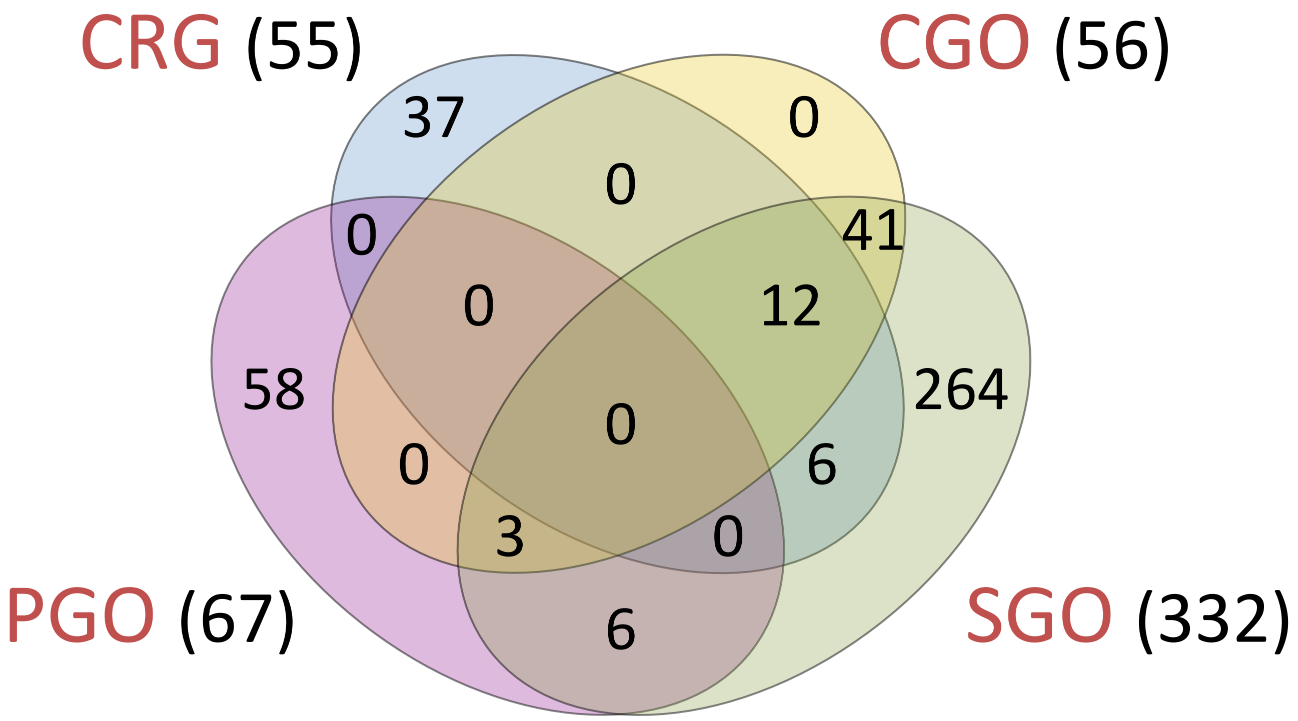

Venn-diagrams showing the numbers of genes reported in the literature as cold responsive genes (CRG), and of genes annotated as involved in either cold response (CGO), photosynthesis (PGO) and general stress responses (SGO). Total numbers for each functional class are given in brackets. Overlapping areas indicate genes common to two or more functional classes.
