# Supplementary material for: Rates of evolution in stress-related genes are associated with habitat preference in two Cardamine lineages
Source: BMC Evol Biol. 2012 Jan 18;12:7. doi: 10.1186/1471-2148-12-7 (PMC3398273; doi:10.1186/1471-2148-12-7)
Supplement: Additional file 2 — Correlation between substitution rate and gene length. Plot showing the correlation between the length of the A. thaliana orthogous gene and the substitution rate in C. impatiens and C. resedifolia genes. [file 1471-2148-12-7-S2.PDF]

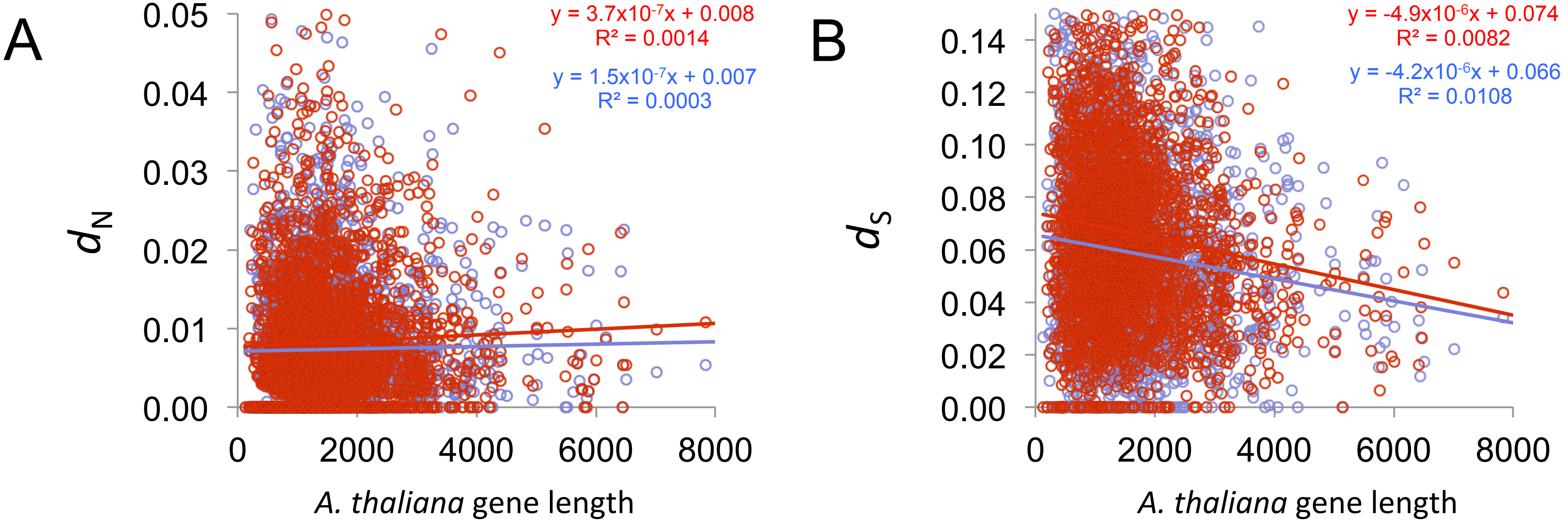

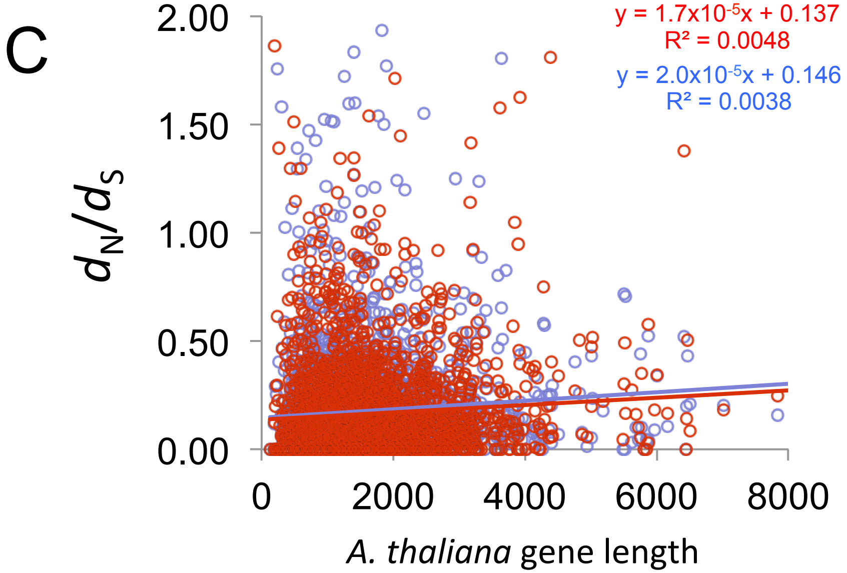

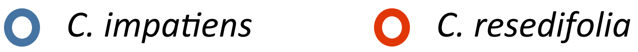

Correlation between the length of the *A. thaliana* orthogous gene and (A) the rate of non-synonymous substitutio, d_N, (B) the rate of synonymous substitution, d_S, and (C) the ratio d_N/d_S, in *C. impatiens* and *C. resedifolia* genes.
