# Supplementary material for: Rates of evolution in stress-related genes are associated with habitat preference in two Cardamine lineages
Source: BMC Evol Biol. 2012 Jan 18;12:7. doi: 10.1186/1471-2148-12-7 (PMC3398273; doi:10.1186/1471-2148-12-7)
Supplement: Additional file 4 — Number of genes identified by likelihood ratio tests that compared branch, site and branch-site codon substitution models. Number of genes identified (at decreasing probabilities thresholds) as putative targets of positive selection by likelihood ratio tests based on branch models (B tests, Table S4.1), site models (S tests, Table S4.2), and branch-site models (BS tests, Table S4.3). [file 1471-2148-12-7-S4.DOC]

## Additional file 4

**Number of genes identified by likelihood ratio tests that compared branch, site and branch-site codon substitution models.**

**Table S4.1. Number of genes identified as putative targets for differential evolution along the phylogeny, using the *branch* codon substitution models.**

| ***P* threshold ^a^** | 0.001 | | |  | 0.0005 | | |  | 0.00025 | | |  | 0.0001 | | |
| --- | --- | --- | --- | --- | --- | --- | --- | --- | --- | --- | --- | --- | --- | --- | --- |
| **Test ^b^** | B | B*_Ci_* | B*_Cr_* |  | B | B*_Ci_* | B*_Cr_* |  | B | B*_Ci_* | B*_Cr_* |  | B | B*_Ci_* | B*_Cr_* |
| **FDR ^c^** | 0.34 | 0.30 | 0.23 |  | 0.34 | 0.30 | 0.22 |  | 0.08 | 0.08 | 0.20 |  | 0.08 | 0.08 | 0.19 |
| *Total* | *8* | *11* | *8* |  | *3* | *5* | *3* |  | *2* | *3* | *1* |  | *2* | *1* | *1* |
| CRG | 0 | 0 | 0 |  | 0 | 0 | 0 |  | 0 | 0 | 0 |  | 0 | 0 | 0 |
| CGO | 0 | 0 | 0 |  | 0 | 0 | 0 |  | 0 | 0 | 0 |  | 0 | 0 | 0 |
| PGO | 0 | 0 | 0 |  | 0 | 0 | 0 |  | 0 | 0 | 0 |  | 0 | 0 | 0 |
| SGO | 2 | 2 | 0 |  | 1 | 2 | 0 |  | 1 | 1 | 0 |  | 1 | 1 | 0 |

^a^ Probability threshold.

^b^ Type of Likelihood Ratio Test. Test B compared the likelihoods of models M0 and M0’. Tests were also performed setting as foreground braches either the *C. impatiens* lineage (B*_Ci_*) or the *C. resedifolia* lineages (B*_Cr_*; see main text for details).

^c^ False discovery rate associated to the probability threshold.

CRG = cold responsive genes; CGO = genes involved in cold acclimation; PGO = genes involved in photosynthesis; SGO = genes broadly involved in stress resistance (see main text for details).

**Table S4.2. Number of genes identified as putative targets of positive selection using the site codon substitution models.**

| ***P* threshold ^a^** | 0.001 | |  | 0.0005 | |  | 0.00025 | |  | 0.0001 | |
| --- | --- | --- | --- | --- | --- | --- | --- | --- | --- | --- | --- |
| **Test ^b^** | S_21_ | S_87_ |  | S_21_ | S_87_ |  | S_21_ | S_87_ |  | S_21_ | S_87_ |
| **FDR ^c^** | 0.34 | 0.27 |  | 0.20 | 0.22 |  | 0.12 | 0.17 |  | 0.12 | 0.10 |
| *Total* | *5* | *6* |  | *4* | *5* |  | *2* | *3* |  | *2* | *2* |
| CRG | 0 | 0 |  | 0 | 0 |  | 0 | 0 |  | 0 | 0 |
| CGO | 0 | 0 |  | 0 | 0 |  | 0 | 0 |  | 0 | 0 |
| PGO | 0 | 0 |  | 0 | 0 |  | 0 | 0 |  | 0 | 0 |
| SGO | 2 | 2 |  | 1 | 1 |  | 0 | 1 |  | 0 | 0 |

^a^ Probability threshold.

^b^ Type of Likelihood Ratio Test. Test S_21_ compared the likelihoods of models M1a and M2a. Test S_87_ compared the likelihoods of models M7 and M8 (see main text for details).

^c^ False discovery rate associated to the probability threshold.

CRG = cold responsive genes; CGO = genes involved in cold acclimation; PGO = genes involved in photosynthesis; SGO = genes broadly involved in stress resistance (see main text for details).

**Table S4.3. Number of genes identified as putative targets of positive selection using the *branch*-*site* codon substitution models.**

| ***P* threshold ^a^** | 0.001 | |  | 0.0005 | |  | 0.00025 | |  | 0.0001 | |
| --- | --- | --- | --- | --- | --- | --- | --- | --- | --- | --- | --- |
| **Test ^b^** | BS*_Ci_* | BS*_Cr_* |  | BS*_Ci_* | BS*_Cr_* |  | BS*_Ci_* | BS*_Cr_* |  | BS*_Ci_* | BS*_Cr_* |
| **FDR ^c^** | 0.50 | 0.24 |  | 0.004 | 0.18 |  | 0.004 | 0.11 |  | 0.004 | 0.08 |
| *Total* | *3* | *11* |  | *1* | *7* |  | *1* | *3* |  | *1* | *2* |
| CRG | 0 | 0 |  | 0 | 0 |  | 0 | 0 |  | 0 | 0 |
| CGO | 1 | 0 |  | 0 | 0 |  | 0 | 0 |  | 0 | 0 |
| PGO | 0 | 0 |  | 0 | 0 |  | 0 | 0 |  | 0 | 0 |
| SGO | 1 | 4 |  | 0 | 2 |  | 0 | 1 |  | 0 | 1 |

^a^ Probability threshold.

^b^ Type of Likelihood Ratio Test: either the *C. impatiens* lineage (BS*_Ci_*) or the *C. resedifolia* lineage (BS*_Cr_*) were set as foreground branches (see main text for details).

^c^ False discovery rate associated to the probability threshold.

CRG = cold responsive genes; CGO = genes involved in cold acclimation; PGO = genes involved in photosynthesis; SGO = genes broadly involved in stress resistance (see main text for details).
