# Supplementary material for: Rates of evolution in stress-related genes are associated with habitat preference in two Cardamine lineages
Source: BMC Evol Biol. 2012 Jan 18;12:7. doi: 10.1186/1471-2148-12-7 (PMC3398273; doi:10.1186/1471-2148-12-7)
Supplement: Additional file 5 — Description of the top ten genes identified by likelihood ratio tests that compared various codon substitution models. Top ten genes identified as putative targets of positive selection by likelihood ratio tests based on branch models (B tests, Tables S5.1-S5.3), site models (S tests, Tables S5.4-S5.5), and branch-site models (BS tests, Tables S5.6-S5.7). [file 1471-2148-12-7-S5.DOC]

## Additional file 5

**Description of the top ten genes identified by likelihood ratio tests that compared various codon substitution models**

**Table S5.1. Top ten genes identified by a likelihood ratio test that compared the *branch* codon substitution models M0 and M0’.**

| **Gene** | **Description** | **Function** | ***P* ^a^** | ***q* ^b^** |
| --- | --- | --- | --- | --- |
| AT5G26830 | threonyl-tRNA synthetase / threonine-tRNA ligase (THRRS) | cell wall; membrane; plasma membrane; ATP binding; ligase activity, forming aminoacyl-tRNA and related compounds; nucleotide binding; aminoacyl-tRNA ligase activity; threonine-tRNA ligase activity; tRNA aminoacylation for protein translation; threonyl-tRNA aminoacylation; chloroplast; mitochondrion | 2×10^-6^ | 0.0049 |
| AT1G71040 ^c^ | LPR2 (Low Phosphate Root2); copper ion binding / oxidoreductase | cell wall; membrane; copper ion binding; oxidoreductase activity; cellular response to phosphate starvation; meristem maintenance | 5×10^-5^ | 0.0769 |
| AT3G15470 | WD-40 repeat family protein | heterotrimeric G-protein complex; signal transducer activity; signal transduction | 0.0004 | 0.3398 |
| AT3G52370 | FLA15 (FASCICLIN-LIKE ARABINOGALACTAN PROTEIN 15 PRECURSOR) | endomembrane system; cell adhesion | 0.0006 | 0.3398 |
| AT1G71920 | histidinol-phosphate aminotransferase, putative | chloroplast; catalytic activity; histidinol-phosphate transaminase activity; transferase activity, transferring nitrogenous groups; biosynthetic process; histidine biosynthetic process; pyridoxal phosphate binding | 0.0006 | 0.3398 |
| AT1G52710 | cytochrome c oxidase-related | biological process; mitochondrial envelope; cytochrome-c oxidase activity | 0.0008 | 0.3398 |
| AT3G48000 | ALDH2B4 (ALDEHYDE DEHYDROGENASE 2B4); 3-chloroallyl aldehyde dehydrogenase/ ATP binding | chloroplast; mitochondrion; ATP binding; response to cadmium ion; 3-chloroallyl aldehyde dehydrogenase activity; aldehyde dehydrogenase (NAD) activity | 0.0009 | 0.3398 |
| AT3G26520 ^c^ | TIP2 (TONOPLAST INTRINSIC PROTEIN 2); water | chloroplast; membrane; plasma membrane; vacuole; defense response to bacterium; water channel activity; response to salt stress; transport; water homeostasis | 0.0009 | 0.3398 |
| AT1G08590 | CLAVATA1 receptor kinase (CLV1) | protein amino acid phosphorylation; endomembrane system; kinase activity | 0.0011 | 0.3566 |
| AT5G57280 | methyltransferase | methyltransferase activity; metabolic process | 0.0016 | 0.4413 |

^a^ Likelihood Ratio Tests probability.

^b^ q-value.

^c^ gene of the SGO functional class.

**Table S5.2. Top ten genes identified by a likelihood ratio test that compared the *branch* codon substitution models M0 and M0’ with *C. impatiens* lineage as foreground branch.**

| **Gene** | **Description** | **Function** | ***P* ^a^** | ***q* ^b^** | $\omega_{FB}$**^c^** | $\omega_{R}$**^d^** |
| --- | --- | --- | --- | --- | --- | --- |
| AT1G71040 ^e^ | LPR2 (Low Phosphate Root2); copper ion binding / oxidoreductase | cell wall; membrane; copper ion binding; oxidoreductase activity; cellular response to phosphate starvation; meristem maintenance | 0.0001 | 0.1865 | 0.096 | 0.392 |
| AT5G62680 | proton-dependent oligopeptide transport (POT) family protein | plasma membrane; oligopeptide transport; transporter activity | 0.0002 | 0.1988 | 0.016 | 0.262 |
| AT5G26830 | threonyl-tRNA synthetase / threonine-tRNA ligase (THRRS) | cell wall; membrane; plasma membrane; ATP binding; ligase activity, forming aminoacyl-tRNA and related compounds; nucleotide binding; aminoacyl-tRNA ligase activity; threonine-tRNA ligase activity; tRNA aminoacylation for protein translation; threonyl-tRNA aminoacylation; chloroplast; mitochondrion | 0.0002 | 0.1988 | 0.255 | 0.023 |
| AT1G55210 ^c^ | disease resistance response | endomembrane system; molecular function; defense response; lignan biosynthetic process | 0.0004 | 0.2219 | 0.026 | 0.316 |
| AT5G17170 | ENH1 (enhancer of sos3-1); electron carrier; metal ion binding / protein binding | chloroplast; chloroplast thylakoid membrane; electron carrier activity; protein binding; chloroplast envelope; metal ion binding | 0.0004 | 0.2219 | 0.024 | 999 |
| AT4G25570 | ACYB-2; carbon-monoxide oxygenase | chloroplast; vacuole; integral to membrane; carbon-monoxide oxygenase activity | 0.0007 | 0.2251 | 0.392 | 0.0001 |
| AT5G27470 | seryl-tRNA synthetase / serine-tRNA ligase | cytosol; serine-tRNA ligase activity; seryl-tRNA aminoacylation; ATP binding; nucleotide binding; aminoacyl-tRNA ligase activity; response to cadmium ion | 0.0007 | 0.2251 | 0.0001 | 0.235 |
| AT1G76730 | 5-formyltetrahydrofolate cyclo-ligase family protein | chloroplast; ATP binding; folic acid and derivative biosynthetic process; 5-formyltetrahydrofolate cyclo-ligase activity; catalytic activity; metabolic process | 0.0008 | 0.2251 | 0.059 | 999 |
| AT5G26280 | meprin and TRAF homology domain-containing protein | molecular function; biological process; membrane | 0.0009 | 0.2251 | 0.206 | 1.512 |
| AT1G11580 | enzyme inhibitor; pectinesterase | cell wall; plasma membrane; enzyme inhibitor activity; pectinesterase activity; cell wall modification; plant-type cell wall | 0.0009 | 0.2251 | 0.023 | 0.254 |

^a^ Likelihood Ratio Tests probability.

^b^ q-value.

^c^ ω estimated for the focal branch.

^d^ ω estimated for the rest of the phylogenetic tree.

^e^ gene of the SGO functional class.

**Table S5.3. Top ten genes identified by a likelihood ratio test that compared the *branch* codon substitution models M0 and M0’ with *C. resedifolia* lineage as foreground branch.**

| **Gene** | **Description** | **Function** | ***P* ^a^** | ***q* ^b^** | $\omega_{FB}$**^c^** | $\omega_{R}$**^d^** |
| --- | --- | --- | --- | --- | --- | --- |
| AT2G25840 | OVA4 (ovule abortion 4); ATP binding / aminoacyl-tRNA ligase/ nucleotide binding / tryptophan-tRNA ligase | chloroplast; ATP binding; nucleotide binding; aminoacyl-tRNA ligase activity; tryptophan-tRNA ligase activity; N-terminal protein myristoylation; mitochondrion; tRNA aminoacylation for protein translation; ovule development | 3×10^-5^ | 0.0830 | 0.769 | 0.0001 |
| AT5G62190 | PRH75; ATP-dependent helicase/ DEAD/H-box RNA helicase binding | nucleolus; nucleus; ATP-dependent helicase activity; RNA metabolic process; DEAD/H-box RNA helicase binding | 0.0003 | 0.3006 | 0.376 | 0.017 |
| AT5G26830 | threonyl-tRNA synthetase / threonine-tRNA ligase (THRRS) | cell wall; membrane; plasma membrane; ATP binding; ligase activity, forming aminoacyl-tRNA and related compounds; nucleotide binding; aminoacyl-tRNA ligase activity; threonine-tRNA ligase activity; tRNA aminoacylation for protein translation; threonyl-tRNA aminoacylation; chloroplast; mitochondrion | 0.0004 | 0.3006 | 0.258 | 0.034 |
| AT5G62930 | GDSL-motif lipase/hydrolase family protein | hydrolase activity; hydrolase activity, acting on ester bonds; lipid metabolic process; cellular component; carboxylesterase activity | 0.0008 | 0.3006 | 0.056 | 1.137 |
| AT5G17270 | tetratricopeptide repeat (TPR)-containing protein | binding; cellular component; biological process | 0.0008 | 0.3006 | 0.134 | 0.741 |
| AT3G18780 | ACT2 (ACTIN 2); structural constituent of cytoskeleton | membrane; nucleus; cytoplasm; structural constituent of cytoskeleton; response to high light intensity; response to cytokinin stimulus; response to red light; root epidermal cell differentiation; response to far red light; root hair elongation; root hair cell tip growth | 0.0008 | 0.3006 | 0.000 | 0.093 |
| AT4G29220 | PFK1 (PHOSPHOFRUCTOKINASE 1); 6-phosphofructokinase | 6-phosphofructokinase complex; cytosol; plasma membrane; 6-phosphofructokinase activity; glycolysis | 0.0009 | 0.3006 | 0.240 | 0.0001 |
| AT5G66050 | Unknown protein | Unknown function | 0.0010 | 0.3006 | 0.400 | 0.0001 |
| AT1G15910 | XH/XS domain-containing protein / XS zinc finger domain-containing protein | cellular component; molecular function; biological process | 0.0011 | 0.3006 | 0.807 | 0.097 |
| AT3G14900 | Unknown protein | Unknown function | 0.0015 | 0.3388 | 0.018 | 0.193 |

^a^ Likelihood Ratio Tests probability.

^b^ q-value.

^c^ ω estimated for the focal branch.

^d^ ω estimated for the rest of the phylogenetic tree.

^e^ gene of the SGO functional class.

**Table S5.4. Top ten genes identified by a likelihood ratio test that compared the *site* codon substitution models M1a and M2a.**

| **Gene** | **Description** | **Function** | ***P* ^a^** | ***q* ^b^** | ***p*_2_ ^c^** | $\omega_{2}$**^d^** |
| --- | --- | --- | --- | --- | --- | --- |
| AT5G06980 | unknown protein | cellular component; molecular function; biological process | 3×10^-6^ | 0.0089 | 0.045 | 115.6 |
| AT4G17520 | nuclear RNA-binding protein, putative | peroxisome; nucleus; RNA binding; biological process | 8×10^-5^ | 0.1223 | 0.057 | 13.3 |
| AT2G31610 ^e^ | 40S ribosomal protein S3 (RPS3A) | chloroplast; membrane; plasma membrane; vacuole; response to salt stress; cytosolic ribosome; structural constituent of ribosome; translation; response to abiotic stimulus; cytosolic small ribosomal subunit | 0.0003 | 0.1986 | 0.024 | 17.6 |
| AT1G49750 | leucine-rich repeat family protein | chloroplast; protein binding | 0.0003 | 0.1986 | 0.138 | 5.6 |
| AT1G54040 ^e^ | ESP (EPITHIOSPECIFIER PROTEIN); enzyme regulator | nucleus; enzyme regulator activity; glucosinolate catabolic process; response to jasmonic acid stimulus; defense response to bacterium; leaf senescence | 0.0006 | 0.3356 | 0.041 | 14.5 |
| AT4G23850 | long-chain-fatty-acid-CoA ligase / long-chain acyl-CoA synthetase | plasma membrane; catalytic activity; fatty acid biosynthetic process | 0.0011 | 0.5280 | 0.006 | 375.2 |
| AT5G07030 | aspartic-type endopeptidase | cell wall; plant-type cell wall; proteolysis | 0.0013 | 0.5280 | 0.009 | 999.0 |
| AT2G44060 ^e^ | late embryogenesis abundant family protein / LEA family protein | membrane; plasma membrane; response to cadmium ion; molecular function; response to desiccation; embryonic development ending in seed dormancy | 0.0016 | 0.5824 | 0.011 | 63.6 |
| AT2G19270 | NA | Unknown function | 0.0018 | 0.5824 | 0.017 | 258.7 |
| AT3G13930 | dihydrolipoamide S-acetyltransferase, putative | chloroplast; mitochondrion; acyltransferase activity; dihydrolipoyllysine-residue acetyltransferase activity; protein binding; metabolic process; pyruvate metabolic process; chloroplast envelope | 0.0034 | 0.9738 | 0.011 | 17.4 |

^a^ Likelihood Ratio Tests probability.

^b^ q-value.

^c^ Proportion of sites under positive selection estimated by model M2a.

^d^ ω estimated for the sites under positive selection by model M2a.

^e^ gene of the SGO functional class.

**Table S5.5. Top ten genes identified by a likelihood ratio test that compared the *site* codon substitution models M7 and M8.**

| **Gene** | **Description** | **Function** | ***P* ^a^** | ***q* ^b^** | ***p*_1_ ^c^** | $\omega_{s}$**^d^** |
| --- | --- | --- | --- | --- | --- | --- |
| AT5G06980 | unknown protein | cellular component; molecular function; biological process | 2×10^-6^ | 0.0060 | 0.045 | 999.0 |
| AT4G17520 | nuclear RNA-binding protein, putative | peroxisome; nucleus; RNA binding; biological process | 7×10^-5^ | 0.1000 | 0.056 | 13.5 |
| AT2G31610 ^e^ | 40S ribosomal protein S3 (RPS3A) | chloroplast; membrane; plasma membrane; vacuole; response to salt stress; cytosolic ribosome; structural constituent of ribosome; translation; response to abiotic stimulus; cytosolic small ribosomal subunit | 0.0002 | 0.1652 | 0.024 | 17.7 |
| AT1G49750 | leucine-rich repeat family protein | chloroplast; protein binding | 0.0003 | 0.1981 | 0.142 | 5.6 |
| AT4G23850 | long-chain-fatty-acid-CoA ligase / long-chain acyl-CoA synthetase | plasma membrane; catalytic activity; fatty acid biosynthetic process | 0.0004 | 0.2189 | 0.041 | 14.6 |
| AT1G54040 ^e^ | ESP (EPITHIOSPECIFIER PROTEIN); enzyme regulator | nucleus; enzyme regulator activity; glucosinolate catabolic process; response to jasmonic acid stimulus; defense response to bacterium; leaf senescence | 0.0005 | 0.2679 | 0.006 | 496.7 |
| AT5G07030 | aspartic-type endopeptidase | cell wall; plant-type cell wall; proteolysis | 0.0011 | 0.4201 | 0.009 | 999.0 |
| AT2G44060 ^e^ | late embryogenesis abundant family protein / LEA family protein | membrane; plasma membrane; response to cadmium ion; molecular function; response to desiccation; embryonic development ending in seed dormancy | 0.0011 | 0.4201 | 0.011 | 63.6 |
| AT4G29350 | PFN2 (PROFILIN 2); actin binding / protein binding | chloroplast; plasma membrane; actin cytoskeleton; cytoplasm; actin binding; protein binding; actin polymerization or depolymerization; cytoskeleton organization | 0.0013 | 0.4298 | 0.017 | 259.2 |
| AT4G25130 | peptide methionine sulfoxide reductase, putative | chloroplast; peptide-methionine-(S)-S-oxide reductase activity; protein modification process; chloroplast stroma; oxidoreductase activity, acting on sulfur group of donors, disulfide as acceptor; protein metabolic process | 0.0015 | 0.4309 | 0.011 | 17.4 |

^a^ Likelihood Ratio Tests probability.

^b^ q-value.

^c^ Proportion of sites under positive selection estimated by model M8.

^d^ ω estimated for the sites under positive selection by model M8.

^e^ gene of the SGO functional class.

**Table S5.6. Top ten genes identified by the *branch-site* model A, test 2, with *C. impatiens* lineage as foreground branch.**

| **Gene** | **Description** | **Function** | ***P* ^a^** | ***q* ^b^** | ***p*_2a_ *+p*_2b_ ^c^** | $\omega_{2}$**^d^** |
| --- | --- | --- | --- | --- | --- | --- |
| AT4G17520 | nuclear RNA-binding protein, putative | peroxisome; nucleus; RNA binding; biological process | 1×10^-6^ | 0.0035 | 0.029 | 125.0 |
| AT4G01150 | unknown protein | chloroplast; thylakoid; chloroplast thylakoid membrane; chloroplast envelope; plastoglobule; molecular function; biological process | 0.0005 | 0.5023 | 0.008 | 999.0 |
| AT3G01500 ^e^ | CA1 (CARBONIC ANHYDRASE 1); carbonate dehydratase/ zinc ion binding | chloroplast; chloroplast stroma; membrane; thylakoid; chloroplast thylakoid membrane; carbonate dehydratase activity; zinc ion binding; chloroplast envelope; apoplast; defense response to bacterium; carbon utilization; response to cold; stromule | 0.0005 | 0.5023 | 0.012 | 999.0 |
| AT1G05870 | unknown protein | N-terminal protein myristoylation; cellular component; molecular function | 0.0014 | 0.8102 | 0.046 | 999.0 |
| AT1G59870 ^e^ | PEN3 (PENETRATION 3); ATPase, coupled to transmembrane movement of substances / cadmium ion transmembrane transporter | mitochondrion; plasma membrane; chloroplast envelope; cadmium ion transmembrane transporter activity; multidrug transport; systemic acquired resistance; defense response to fungus and bacterium; indole glucosinolate catabolic process; callose deposition in cell wall during defense response | 0.0014 | 0.8102 | 0.028 | 76.8 |
| AT4G26300 | emb1027 (embryo defective 1027); ATP binding / aminoacyl-tRNA ligase/ arginine-tRNA ligase/ nucleotide binding | chloroplast; ATP binding; arginine-tRNA ligase activity; nucleotide binding; aminoacyl-tRNA ligase activity; mitochondrion; arginyl-tRNA aminoacylation; embryonic development ending in seed dormancy | 0.0030 | 1.0000 | 0.015 | 85.7 |
| AT2G30280 | unknown protein | cellular component | 0.0034 | 1.0000 | 0.020 | 999.0 |
| AT1G49750 | leucine-rich repeat family protein | chloroplast; protein binding | 0.0042 | 1.0000 | 0.042 | 42.0 |
| AT2G20930 | NA | intracellular; ER to Golgi vesicle-mediated transport; molecular function | 0.0054 | 1.0000 | 0.058 | 67.2 |
| AT1G09310 | unknown protein | apoplast; biological process | 0.0070 | 1.0000 | 0.032 | 123.9 |

^a^ Likelihood Ratio Tests probability.

^b^ q-value.

^c^ Proportion of sites under positive selection estimated in the foreground branch by the *branch-site* model A.

^d^ ω estimated for the sites under positive selection in the foreground branch by the *branch-site* model A.

^e^ gene of the SGO functional class.

**Table S5.7. Top ten genes identified by the *branch-site* model A, test 2, with *C. resedifolia* lineage as foreground branch.**

| **Gene** | **Description** | **Function** | ***P* ^a^** | ***q* ^b^** | ***p*_2a_ *+p*_2b_ ^c^** | $\omega_{2}$**^d^** |
| --- | --- | --- | --- | --- | --- | --- |
| AT5G20900 | JAZ12 (JASMONATE-ZIM-DOMAIN PROTEIN 12) | cellular component; molecular function; biological process | 3×10^-6^ | 0.0081 | 0.060 | 395.6 |
| AT1G54040 ^e^ | ESP (EPITHIOSPECIFIER PROTEIN); enzyme regulator | nucleus; enzyme regulator activity; glucosinolate catabolic process; response to jasmonic acid stimulus; defense response to bacterium; leaf senescence | 0.0001 | 0.0777 | 0.058 | 37.4 |
| AT3G52910 | AtGRF4 (GROWTH-REGULATING FACTOR 4); transcription activator | nucleus; transcription activator activity; leaf development | 0.0001 | 0.1065 | 0.079 | 15.1 |
| AT1G14610 | TWN2 (TWIN 2); ATP binding / aminoacyl-tRNA ligase/ nucleotide binding / valine-tRNA ligase | ATP binding; nucleotide binding; aminoacyl-tRNA ligase activity; valine-tRNA ligase activity; chloroplast; mitochondrion; tRNA aminoacylation for protein translation; embryonic development ending in seed dormancy | 0.0003 | 0.1753 | 0.008 | 99.6 |
| AT1G07890 ^e^ | APX1 (ascorbate peroxidase 1); L-ascorbate peroxidase | cell wall; chloroplast; chloroplast stroma; plasma membrane; response to salt stress; response to cadmium ion; cytosol; L-ascorbate peroxidase activity; response to heat; response to reactive oxygen species; embryonic development ending in seed dormancy | 0.0003 | 0.1753 | 0.018 | 999.0 |
| AT3G06130 | heavy-metal-associated domain-containing protein | cell wall; metal ion transport; metal ion binding | 0.0004 | 0.1792 | 0.037 | 75.3 |
| AT1G21680 | NA | plant-type cell wall; plasma membrane; vacuole; molecular function; biological process | 0.0004 | 0.1792 | 0.011 | 636.2 |
| AT1G49750 | leucine-rich repeat family protein | chloroplast; protein binding | 0.0007 | 0.2434 | 0.046 | 22.5 |
| AT1G58200 ^e^ | MSL3 (MscS-LIKE 3); ion channel | membrane; plastid envelope; ion channel activity; response to osmotic stress; plastid organization | 0.0008 | 0.2434 | 0.012 | 44.1 |
| AT1G54270 | EIF4A-2; ATP-dependent helicase/ translation initiation factor | plasma membrane; response to cadmium ion; cytosol; ATP-dependent helicase activity; translation initiation factor activity | 0.0009 | 0.2434 | 0.003 | 735.7 |

^a^ Likelihood Ratio Tests probability.

^b^ q-value.

^c^ Proportion of sites under positive selection estimated in the foreground branch by the *branch-site* model A.

^d^ ω for the sites under positive selection estimated in the foreground branch by the *branch-site* model A.

^e^ gene of the SGO functional class.
