# Supplementary material for: Rates of evolution in stress-related genes are associated with habitat preference in two Cardamine lineages
Source: BMC Evol Biol. 2012 Jan 18;12:7. doi: 10.1186/1471-2148-12-7 (PMC3398273; doi:10.1186/1471-2148-12-7)
Supplement: Additional file 6 — Phylogenetic trees of candidate genes. Phylogenetic trees for the five genes that were putative targets for positive selection (at FDR < 0.20) according to likelihood ratio tests based on the site and branch-site codon substitution models implemented in PAML. [file 1471-2148-12-7-S6.PDF]

Additional File 6

Phylogenetic trees of the fast evolving genes and of their putative orthologues and paralogues .

We used phylogenetic tree analysis to test the possibility that the rates of evolution observed in genes evolving fast along a branch of the phylogeny and in the candidate genes for positive selection were instead the result of a mis-identification of the putative orthologues.
For each candidate gene detected at a false discovery rate threshold of 0.20 (see Table XX), we searched the homologues (and possibly paralogues) in *A. thaliana* and in the two *Cardamine* datasets. BLAST searches identified putative paralogous of six of such genes in at least one among *A. thaliana*, *C. resedifolia* and *C. impatiens*. For these genes we proceeded with the phylogenetic analysis by first searching for the homologues of the *A. thaliana* gene (and, if present, of its paralogues) in other eudicots using BLASTx. Then, sequences of BLAST hits with a reasonable e-value (< 10^{-50}) were aligned using MUSCLE [1], and the subsequent alignment was used to build an unrooted neighbor joining phylogenetic tree using PAUP* [2] (bootstrap values obtained from 1,000 replications). Trees were drawn using Geneious 5.4 [3].

Phylogenetic trees were obtained for the candidate genes AT1G49750 (panel A), AT1G54040 (panel B), AT1G71040 (panel C), AT2G31610 (panel D), AT4G17520 (panel E), and AT5G62680 (panel F).

Sequences of the *A. thaliana, C. impatiens* and *C. resedifolia* orthologues used in our analyses are highlighted in red. Sequences were named using a four letter code identifying the species (see legend below), and followed by either the AGI name of the gene (for *A. thaliana*), the name of the contig (for *C. impatiens and C. resedifolia*), or the unique NCBI gene identifier.

***Alyr** = Arabidopsis lyrata; **Atha** = Arabidopsis thaliana; **Bole** = Brassica oleracea; **Brap** = Brassica rapa; **Cgra** = Capsella grandiflora; **Cimp** = Cardamine impatiens; **Cres** = Cardamine resedifolia; **Ghir** = Gossypium hirsutum; **Gmax** = Glycine max; **Lsat** = Lepidium sativum; **Mtru** = Medicago truncatula; **Npan** = Neslia paniculata; **Ptri** = Populus trichocarpa; **Rcom** = Ricinus communis; **Stub** = Solanum tuberosum; **Thal** = Thellungiella halophila; **Vvin** = Vitis vinifera.*

References

1.Edgar RC: **MUSCLE: multiple sequence alignment with high accuracy and high throughput.** Nucleic Acids Res 2004, **32**:1792-1797.
2.Swofford DL: **PAUP*. Phylogenetic analysis using parsimony (*and other methods). Version 4.** Sinauer Associates, Sunderland, Massachusetts; 2003.
3.Drummond AJ, Ashton B, Buxton S, Cheung M, Cooper A, Duran C, Field M, Heled J, Kearse M, Markowitz S, Moir R, Stones-Havas S, Sturrock S, Thierer T, Wilson A. **Geneious v5.4**, Available from http://www.geneious.com

A

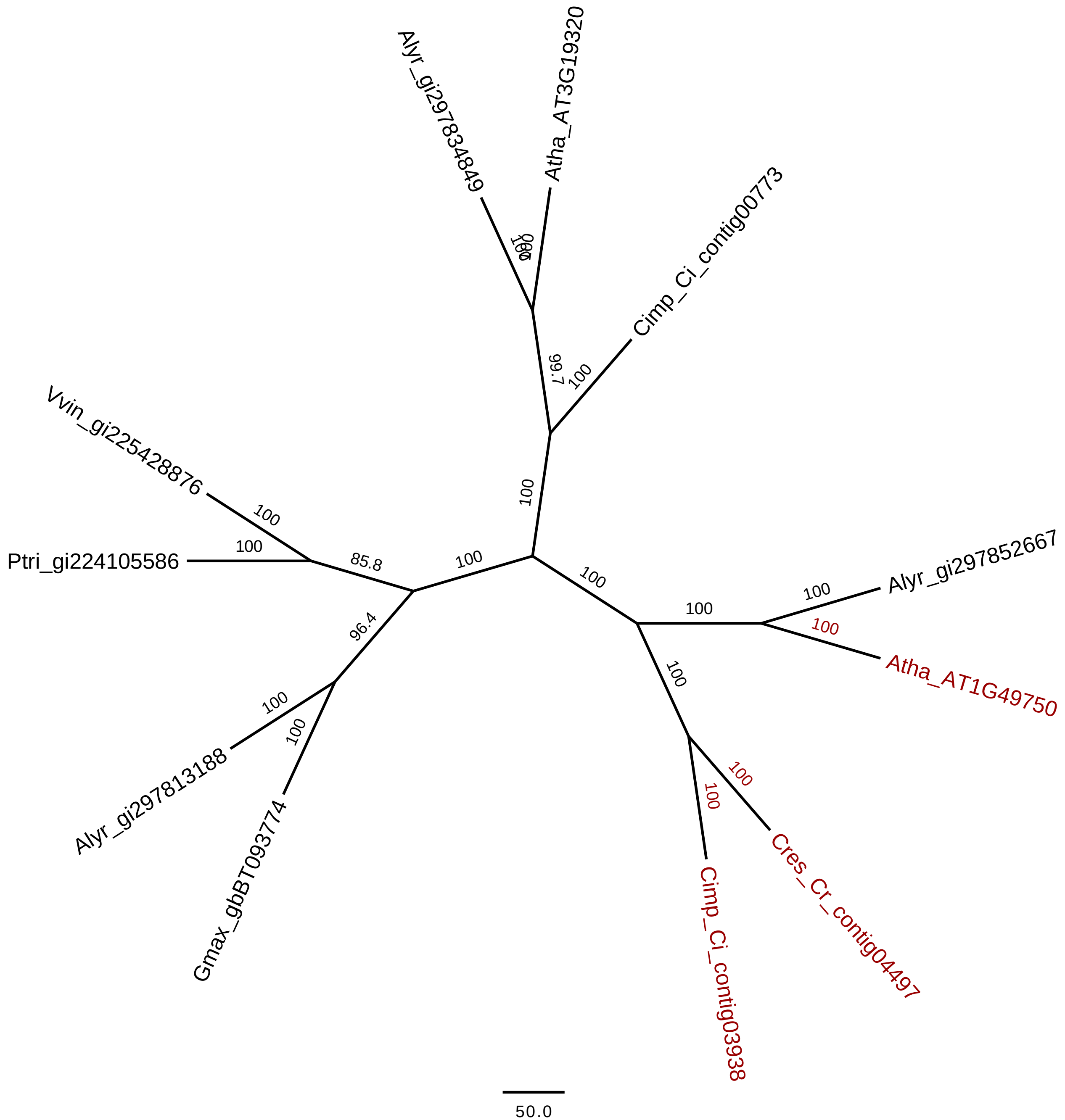
Alyr_gi297834849
Atha_AT3G19320
Cimp_Ci_contig00773
Vvin_gi225428876
Ptri_gi224105586
Alyr_gi297852667
Atha_AT1G49750
Alyr_gi297813188
Gmax_gbBT093774
Cimp_Ci_contig03938
Cres_Cr_contig04497
99.7
100
85.8
96.4
50.0

B

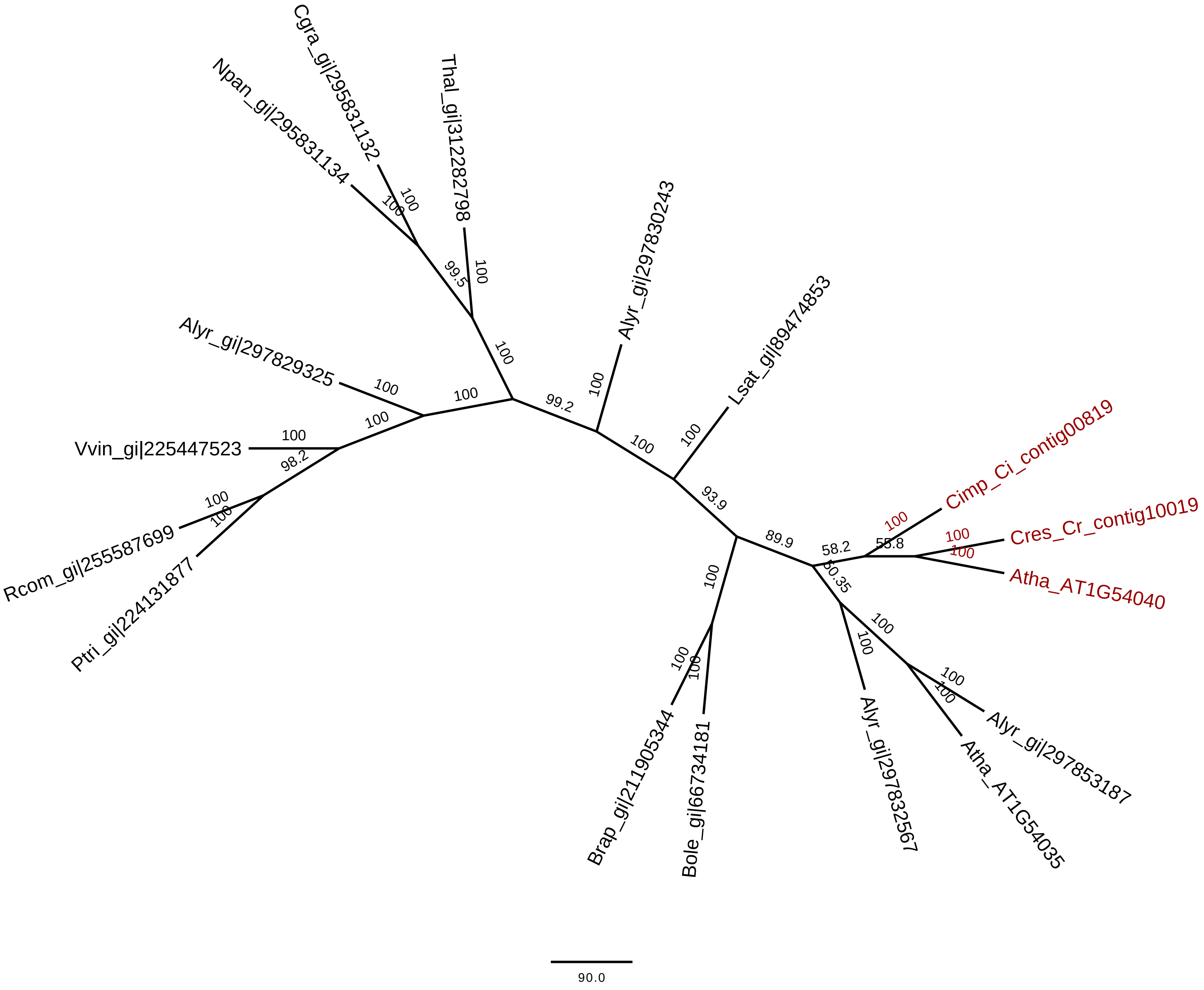
Cgra_gi|295831132
Thal_gi|312282798
Npan_gi|295831134
Alyr_gi|297830243
Lsat_gi|89474853
Alyr_gi|297829325
Vvin_gi|225447523
Rcom_gi|255587699
Ptri_gi|224131877
Cimp_Ci_contig00819
Cres_Cr_contig10019
Atha_AT1G54040
Brap_gi|211905344
Bole_gi|66734181
Alyr_gi|297832567
Atha_AT1G54035
Alyr_gi|297853187
100
99.5
99.2
98.2
93.9
89.9
58.2
55.8
50.35
90.0

C

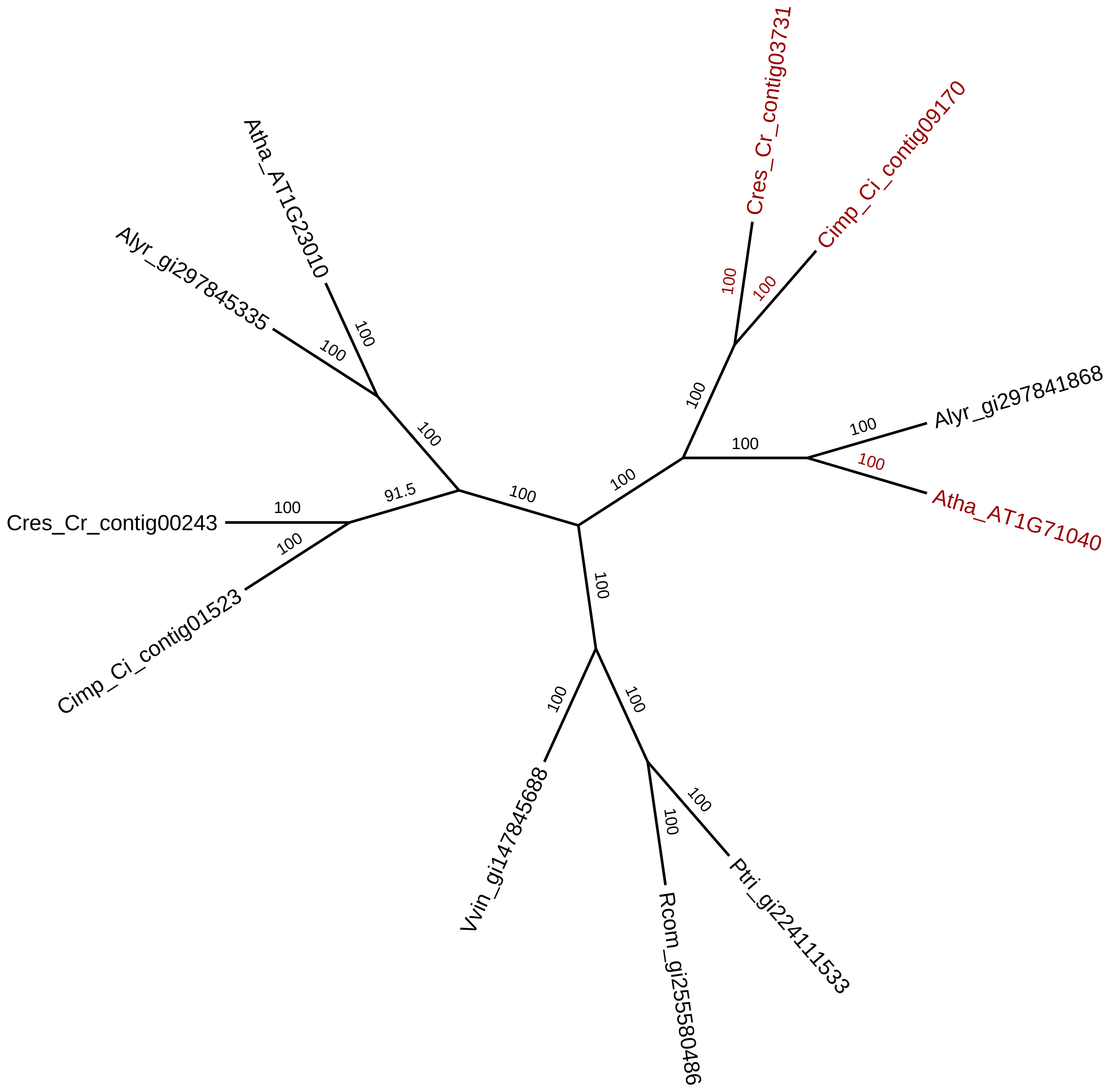
Atha_AT1G23010
Alyr_gi297845335
Cres_Cr_contig00243
Cimp_Ci_contig01523
Cres_Cr_contig03731
Cimp_Ci_contig09170
Alyr_gi297841868
Atha_AT1G71040
Vvin_gi147845688
Rcom_gi255580486
Ptri_gi224111533
100
91.5
60.0

D

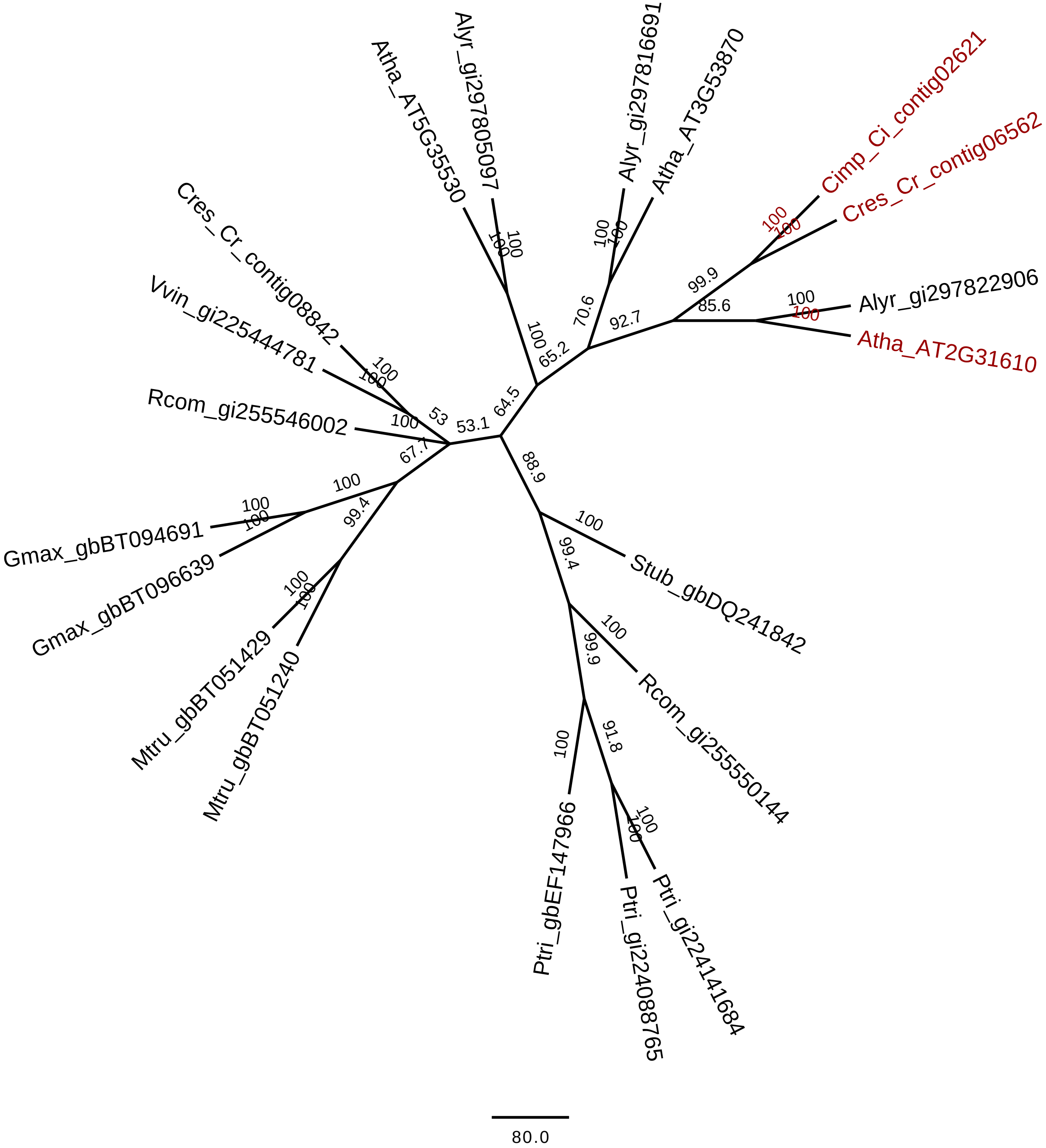

Atha_AT5G35530
Alyr_gi297805097
Alyr_gi297816691
Atha_AT3G53870
Cimp_Ci_contig02621
Cres_Cr_contig06562
Alyr_gi297822906
Atha_AT2G31610
Cres_Cr_contig08842
Vvin_gi225444781
Rcom_gi255546002
Gmax_gbBT094691
Gmax_gbBT096639
Mtru_gbBT051429
Mtru_gbBT051240
Stub_gbDQ241842
Rcom_gi255550144
Ptri_gbEF147966
Ptri_gi224088765
Ptri_gi224141684
80.0

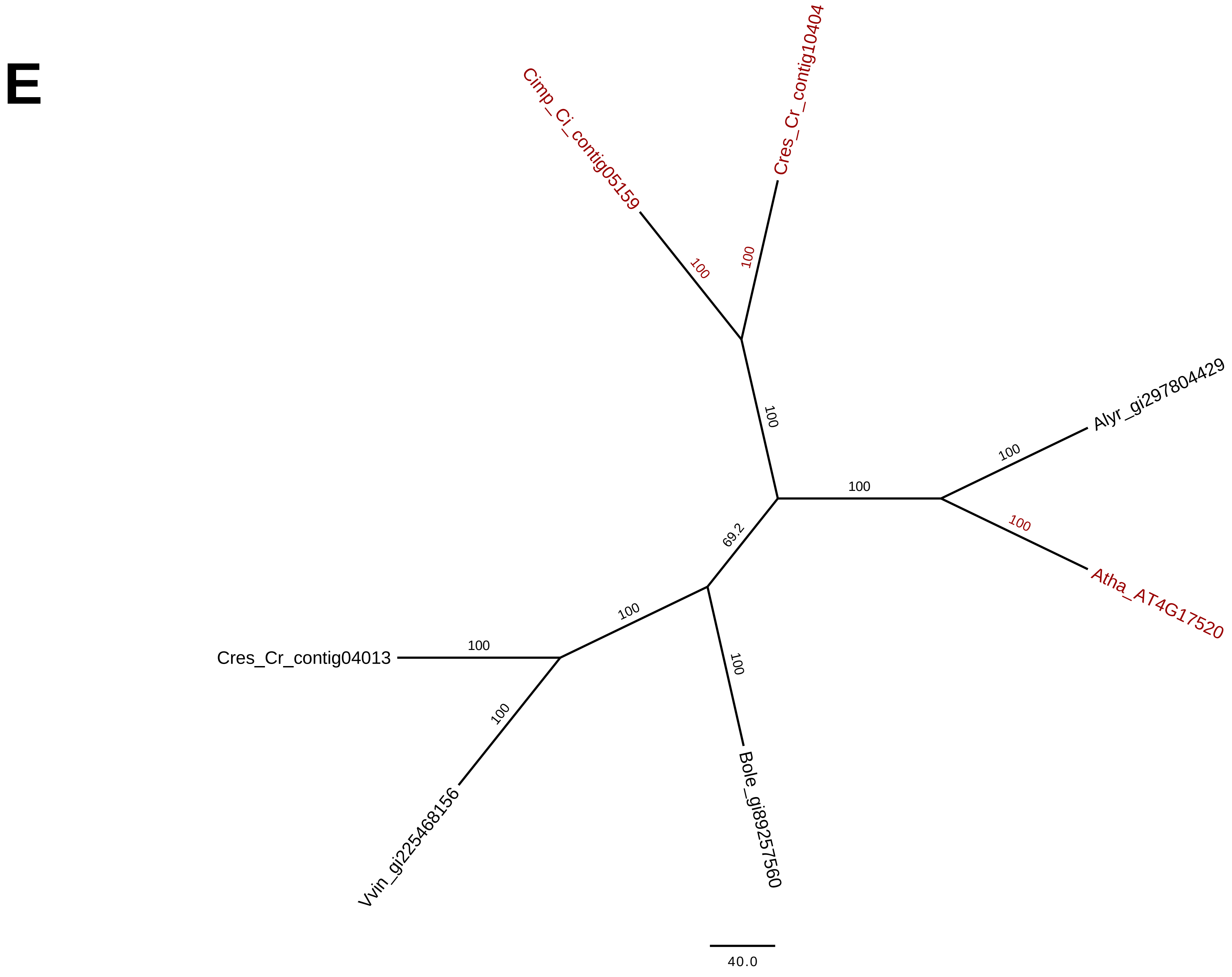
E
Cimp_Ci_contig05159
Cres_Cr_contig10404
Alyr_gi297804429
Atha_AT4G17520
Cres_Cr_contig04013
Vvin_gi225468156
Bole_gi89257560
100
100
100
100
100
100
69.2
100
100
100
100
40.0

F

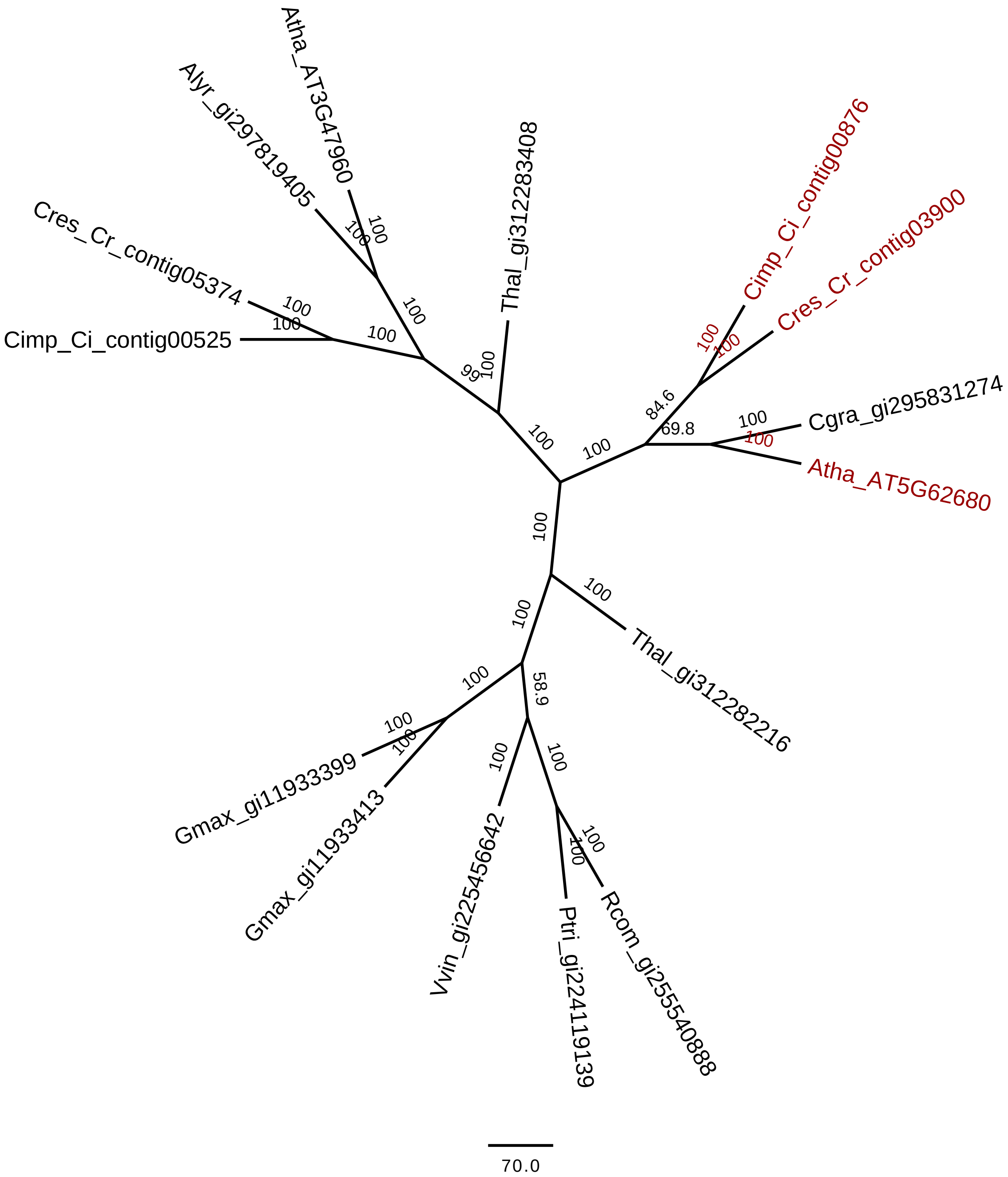
Atha_AT3G47960
Alyr_gi297819405
Thal_gi312283408
Cres_Cr_contig05374
Cimp_Ci_contig00525
Cimp_Ci_contig00876
Cres_Cr_contig03900
Cgra_gi295831274
Atha_AT5G62680
Thal_gi312282216
Gmax_gi11933399
Gmax_gi11933413
Vvin_gi225456642
Ptri_gi224119139
Rcom_gi255540888
70.0
