# Supplementary material for: Rates of evolution in stress-related genes are associated with habitat preference in two Cardamine lineages
Source: BMC Evol Biol. 2012 Jan 18;12:7. doi: 10.1186/1471-2148-12-7 (PMC3398273; doi:10.1186/1471-2148-12-7)
Supplement: Additional file 7 — Correlation between the temporal breadth of expression and levels of selection. Spearman's correlations between levels of selection and both the spatial and temporal breadth of expression. [file 1471-2148-12-7-S7.DOC]

## Additional File 7

**Correlation between the breadth of expression and the ratio *d*_N_/*d*_S_.**

|  | ***C. impatiens*** | | | | |  | ***C. resedifolia*** | | | | |
| --- | --- | --- | --- | --- | --- | --- | --- | --- | --- | --- | --- |
|  |  |  |  | **residuals ^b^** | |  |  |  |  | **residuals ^b^** | |
| **Breadth type ^a^** | **rho ^c^** | ***P* ^c^** |  | **rho ^c^** | ***P* ^c^** |  | **rho ^c^** | ***P* ^c^** |  | **rho ^c^** | ***P* ^c^** |
| Flower development | -0.156 | 5×10^-16^ |  | -0.131 | 1×10^-11^ |  | -0.156 | 4×10^-16^ |  | -0.100 | 2×10^-7^ |
| Leaf development | -0.115 | 3×10^-9^ |  | -0.103 | 1×10^-7^ |  | -0.145 | 4×10^-14^ |  | -0.102 | 1×10^-7^ |
|  |  |  |  |  |  |  |  |  |  |  |  |
| Organs | -0.131 | 1×10^-11^ |  | -0.120 | 5×10^-10^ |  | -0.135 | 2×10^-12^ |  | -0.111 | 9×10^-9^ |
| Organ specificity τ | 0.125 | 1×10^-10^ |  | 0.113 | 6×10^-9^ |  | 0.113 | 4×10^-9^ |  | 0.105 | 5×10^-8^ |
|  |  |  |  |  |  |  |  |  |  |  |  |
| UV-B stress | 0.061 | 0.0016 |  | -0.014 | 0.4817 |  | 0.036 | 0.0619 |  | 0.010 | 0.5891 |
| Salt stress | 0.057 | 0.0035 |  | -0.013 | 0.4978 |  | 0.070 | 0.0003 |  | 0.035 | 0.0731 |
| Osmotic stress | 0.039 | 0.0434 |  | -0.015 | 0.4343 |  | 0.057 | 0.0032 |  | 0.034 | 0.0797 |
| Drought stress | 0.050 | 0.0101 |  | -0.033 | 0.0850 |  | 0.032 | 0.0931 |  | 0.011 | 0.5848 |
| Cold stress | 0.043 | 0.0273 |  | -0.016 | 0.4216 |  | 0.052 | 0.0072 |  | 0.028 | 0.1407 |

^a^ Breadth of expression can be either spatial (i.e., number of tissues in which a gene is expressed) or temporal (when a gene is expressed, during either development or stress exposure). Note that the organ-specificity index τ is inversely correlated to the number (breadth) of organs at which a gene is expressed, namely the larger the index the more a gene has a organ-specific expression pattern.

^b^ Correlations between the residuals of the correlation between *d*_N_/*d*_S_ and the length of the *A. thaliana* gene and the residuals of the correlation between breadth of expression and the length of the *A. thaliana* gene.

^c^ Spearman’s correlation.
