# Supplementary material for: Rates of evolution in stress-related genes are associated with habitat preference in two Cardamine lineages
Source: BMC Evol Biol. 2012 Jan 18;12:7. doi: 10.1186/1471-2148-12-7 (PMC3398273; doi:10.1186/1471-2148-12-7)
Supplement: Additional file 8 — Correlation between rate of molecular evolution and level of gene expression. Mean and maximum expression levels, and Spearman's correlations between such expression levels and levels of selection for the genes included in the four functional classes considered in this study. [file 1471-2148-12-7-S8.DOC]

## Additional File 8

**Correlation between the ratio *d*_N_/*d*_S_ and the level of gene expression.**

|  |  |  |  |  |  |  | **Correlation with *d*_N_/*d*_S_** | | | | |  | **Partial correlation ^e^** | | | | |
| --- | --- | --- | --- | --- | --- | --- | --- | --- | --- | --- | --- | --- | --- | --- | --- | --- | --- |
|  |  |  | **mean (SE) ^c^** | |  |  | ***C. impatiens*** | |  | ***C. resedifolia*** | |  | ***C. impatiens*** | |  | ***C. resedifolia*** | |
|  | **Gene FC ^a^** | ***n* ^b^** | **Genes in FC** | **Genes not in FC** | ***P* ^c^** |  | **rho ^d^** | ***P* ^d^** |  | **rho ^d^** | ***P* ^d^** |  | **rho ^d^** | ***P* ^d^** |  | **rho ^d^** | ***P* ^d^** |
| Average Expression | All genes | 2922 | -- | 8.72 (0.03) | -- |  | -0.249 | 2×10^-39^ |  | -0.297 | 8×10^-57^ |  | -0.220 | 7×10^-31^ |  | -0.270 | 1×10^-46^ |
|  | Cold response (CRG) | 55 | 8.49 (0.21) | 8.73 (0.22) | 0.3710 |  | -0.363 | 0.0071 |  | -0.310 | 0.0239 |  | -0.348 | 0.0103 |  | -0.318 | 0.0207 |
|  | Cold (CGO) | 56 | 10.16 (0.22) | 8.70 (0.21) | 5×10^-10^ |  | -0.199 | 0.1499 |  | -0.251 | 0.0642 |  | -0.140 | 0.3101 |  | -0.187 | 0.1712 |
|  | Photosynthesis (PGO) | 67 | 10.29 (0.21) | 8.69 (0.19) | 6×10^-12^ |  | -0.203 | 0.1071 |  | -0.365 | 0.0028 |  | -0.191 | 0.1306 |  | -0.333 | 0.0070 |
|  | Stress (SGO) | 332 | 9.69 (0.09) | 8.60 (0.09) | 7×10^-31^ |  | -0.221 | 0.0001 |  | -0.209 | 0.0002 |  | -0.182 | 0.0011 |  | -0.194 | 0.0005 |
|  |  |  |  |  |  |  |  |  |  |  |  |  |  |  |  |  |  |
| Maximum Expression | All genes | 2922 | -- | 11.02 (0.03) | -- |  | -0.185 | 2×10^-22^ |  | -0.246 | 7×10^-39^ |  | -0.155 | 6×10^-16^ |  | -0.215 | 7×10^-30^ |
|  | Cold response (CRG) | 55 | 12.51 (0.20) | 10.99 (0.24) | 3×10^-9^ |  | -0.126 | 0.3634 |  | -0.190 | 0.1735 |  | -0.132 | 0.3415 |  | -0.204 | 0.1434 |
|  | Cold (CGO) | 56 | 13.06 (0.24) | 10.98 (0.24) | 6×10^-14^ |  | -0.184 | 0.1820 |  | -0.097 | 0.4816 |  | -0.155 | 0.2617 |  | -0.042 | 0.7599 |
|  | Photosynthesis (PGO) | 67 | 12.97 (0.21) | 10.97 (0.22) | 2×10^-15^ |  | -0.198 | 0.1170 |  | -0.367 | 0.0026 |  | -0.185 | 0.1426 |  | -0.357 | 0.0037 |
|  | Stress (SGO) | 332 | 12.26 (0.10) | 10.86 (0.10) | 3×10^-38^ |  | -0.083 | 0.1396 |  | -0.100 | 0.0740 |  | -0.057 | 0.3093 |  | -0.096 | 0.0855 |

^a^ Gene functional class

^b^ Number of genes.

^c^ Mean average and maximum expression (standard error). For each functional class, the mean value was compared against the mean estimated for genes not in functional class using the non-parametric Wilcoxon test.

^d^ Spearman correlation.

^e^ Partial correlation was estimated between the residuals of the correlation between expression and *A. thaliana* orthologue length and the residuals of the correlation between *d*_N_/*d*_S_ and *A. thaliana* orthologue length.
