# Supplementary material for: Rates of evolution in stress-related genes are associated with habitat preference in two Cardamine lineages
Source: BMC Evol Biol. 2012 Jan 18;12:7. doi: 10.1186/1471-2148-12-7 (PMC3398273; doi:10.1186/1471-2148-12-7)
Supplement: Additional file 9 — Rate of synonymous and non-synonymous substitution in Cardamine genes. II. Mean substitution rate in C. resedifolia and C. impatiens genes included in the four functional classes considered in this study. Comparisons of the values corrected for expression levels. [file 1471-2148-12-7-S9.DOC]

## Additional File 9

**Rate of synonymous, *d*_S_, and non-synonymous, *d*_N_, substitution in four functional classes (FC) of *Cardamine* genes, II.**

|  |  |  | ***C. impatiens*** | | | | | |  | ***C. resedifolia*** | | | | | |  | ***C. resedifolia vs. C. impatiens*** | | | |
| --- | --- | --- | --- | --- | --- | --- | --- | --- | --- | --- | --- | --- | --- | --- | --- | --- | --- | --- | --- | --- |
|  | **Gene FC ^a^** |  | ***n* ^b^** | **mean (SE) ^c^** | ***P*_avX_ ^d^** | ***P*_avX_* ^d^** | ***P*_mxX_ ^d^** | ***P*_mxX_* ^d^** |  | ***n* ^b^** | **mean (SE) ^c^** | ***P*_avX_ ^d^** | ***P*_avX_* ^d^** | ***P*_mxX_ ^d^** | ***P*_mxX_* ^d^** |  | ***P*_avX_ ^d^** | ***P*_avX_* ^d^** | ***P*_mxX_ ^d^** | ***P*_mxX_* ^d^** |
| *d*_N_ | CRG | In FC | 55 | 0.0097 (0.0010) | 0.0015 | 0.0105 | 2×10^-5^ | 0.0001 |  | 55 | 0.0106 (0.0012) | 0.0035 | 0.0210 | 1×10^-6^ | 8×10^-6^ |  | 0.9339 | 1 | 0.6296 | 1 |
|  |  | Not in FC | 2858 | 0.0073 (0.0002) |  |  |  |  |  | 2858 | 0.0082 (0.0002) |  |  |  |  |  | 0.1642 | 1 | 0.0721 | 0.5047 |
|  | CGO | In FC | 56 | 0.0058 (0.0008) | 0.2232 | 0.8928 | 0.4698 | 0.9396 |  | 56 | 0.0056 (0.0007) | 0.2750 | 0.8250 | 0.2921 | 0.8763 |  | 0.8203 | 1 | 0.9714 | 0.9714 |
|  |  | Not in FC | 2857 | 0.0074 (0.0002) |  |  |  |  |  | 2857 | 0.0083 (0.0002) |  |  |  |  |  | 0.1645 | 0.9870 | 0.0807 | 0.4842 |
|  | PGO | In FC | 67 | 0.0067 (0.0011) | 0.3195 | 0.6390 | 0.7764 | 0.7764 |  | 67 | 0.0041 (0.0007) | 0.5600 | 0.5600 | 0.1851 | 0.7404 |  | 0.2252 | 0.9008 | 0.2001 | 0.8004 |
|  |  | Not in FC | 2846 | 0.0074 (0.0002) |  |  |  |  |  | 2846 | 0.0084 (0.0002) |  |  |  |  |  | 0.2227 | 1 | 0.1264 | 0.6320 |
|  | SGO | In FC | 332 | 0.0072 (0.0005) | 0.0076 | 0.0380 | 0.1157 | 0.5785 |  | 332 | 0.0075 (0.0005) | 0.0014 | 0.0112 | 0.0131 | 0.0786 |  | 0.9620 | 1 | 0.7960 | 1 |
|  |  | Not in FC | 2581 | 0.0074 (0.0002) |  |  |  |  |  | 2581 | 0.0084 (0.0002) |  |  |  |  |  | 0.1253 | 1 | 0.0549 | 0.4392 |
|  |  |  |  |  |  |  |  |  |  |  |  |  |  |  |  |  |  |  |  |  |
| *d*_S_ | CRG | In FC | 55 | 0.0687 (0.0044) | 0.0122 | 0.0976 | 0.0530 | 0.3710 |  | 55 | 0.0629 (0.0043) | 0.8412 | 1 | 0.4286 | 1 |  | 0.0723 | 0.5784 | 0.0398 | 0.3184 |
|  |  | Not in FC | 2858 | 0.0590 (0.0007) |  |  |  |  |  | 2858 | 0.0666 (0.0010) |  |  |  |  |  | 0.2872 | 1 | 0.3125 | 1 |
|  | CGO | In FC | 56 | 0.0638 (0.0065) | 0.9081 | 0.9081 | 0.7576 | 1 |  | 56 | 0.0731 (0.0081) | 0.7294 | 1 | 0.8924 | 1 |  | 0.9476 | 0.9476 | 0.9714 | 0.9714 |
|  |  | Not in FC | 2857 | 0.0591 (0.0007) |  |  |  |  |  | 2857 | 0.0664 (0.0010) |  |  |  |  |  | 0.1896 | 1 | 0.1958 | 1 |
|  | PGO | In FC | 67 | 0.0526 (0.0041) | 0.0902 | 0.6314 | 0.0322 | 0.2576 |  | 67 | 0.0710 (0.0069) | 0.7828 | 1 | 0.5774 | 1 |  | 0.4763 | 0.9526 | 0.4459 | 0.8918 |
|  |  | Not in FC | 2846 | 0.0593 (0.0007) |  |  |  |  |  | 2846 | 0.0664 (0.0010) |  |  |  |  |  | 0.1479 | 1 | 0.1467 | 1 |
|  | SGO | In FC | 332 | 0.0620 (0.0021) | 0.2377 | 1 | 0.6164 | 1 |  | 332 | 0.0706 (0.0034) | 0.6359 | 1 | 0.9244 | 0.9244 |  | 0.3738 | 1 | 0.4163 | 1 |
|  |  | Not in FC | 2581 | 0.0588 (0.0008) |  |  |  |  |  | 2581 | 0.0660 (0.0010) |  |  |  |  |  | 0.2915 | 1 | 0.2828 | 1 |
|  |  |  |  |  |  |  |  |  |  |  |  |  |  |  |  |  |  |  |  |  |
| *d*_N_/*d*_S_ | CRG | In FC | 55 | 0.1820 (0.0288) | 0.0725 | 0.2900 | 0.0004 | 0.0028 |  | 54 | 0.2431 (0.0510) | 0.0074 | 0.0444 | 1×10^-6^ | 8×10^-6^ |  | 0.1142 | 0.3426 | 0.0465 | 0.1395 |
|  |  | Not in FC | 2792 | 0.1762 (0.0059) |  |  |  |  |  | 2821 | 0.1616 (0.0044) |  |  |  |  |  | 5×10^-8^ | 3×10^-7^ | 8×10^-9^ | 5×10^-8^ |
|  | CGO | In FC | 55 | 0.1260 (0.0205) | 0.0919 | 0.2757 | 0.1238 | 0.2476 |  | 56 | 0.0957 (0.0132) | 0.2193 | 0.4386 | 0.1188 | 0.3564 |  | 0.7275 | 0.7275 | 0.282 | 0.5640 |
|  |  | Not in FC | 2792 | 0.1773 (0.0059) |  |  |  |  |  | 2819 | 0.1645 (0.0045) |  |  |  |  |  | 3×10^-8^ | 2×10^-7^ | 6×10^-9^ | 4×10^-8^ |
|  | PGO | In FC | 65 | 0.1939 (0.0465) | 0.0287 | 0.1435 | 0.0897 | 0.3588 |  | 66 | 0.0840 (0.0175) | 0.9980 | 0.998 | 0.8281 | 0.8281 |  | 0.6393 | 1 | 0.9981 | 0.9981 |
|  |  | Not in FC | 2782 | 0.1759 (0.0058) |  |  |  |  |  | 2809 | 0.1650 (0.0045) |  |  |  |  |  | 1×10^-8^ | 8×10^-8^ | 2×10^-9^ | 2×10^-8^ |
|  | SGO | In FC | 325 | 0.1531 (0.0134) | 0.0032 | 0.0224 | 0.0293 | 0.1465 |  | 328 | 0.1542 (0.0176) | 0.0006 | 0.0048 | 0.0011 | 0.0066 |  | 0.0242 | 0.0968 | 0.0035 | 0.0140 |
|  |  | Not in FC | 2522 | 0.1793 (0.0063) |  |  |  |  |  | 2547 | 0.1643 (0.0045) |  |  |  |  |  | 3×10^-7^ | 2×10^-6^ | 2×10^-7^ | 1×10^-6^ |

^a^ Gene functional class: CRG = cold responsive genes; CGO = genes involved in cold acclimation; PGO = genes involved in photosynthesis; SGO = genes broadly involved in stress resistance (see main text for details).

^b^ Number of genes.

^c^ Mean and standard error.

^d^ Wilcoxon rank-sum test assessing the difference in substitution rates between genes in FC and genes not in FC for both lineages, and between lineages for both genes in FC and genes not in FC. We report the results for the residuals of the correlation between substitution rates and mean gene expression (*P*_avX_), and between substitution rates and maximum gene expression (*P*_mxX_). *P* values followed by an asterisk are corrected for multiple testing (Holm-Bonferroni method).
