# Supplementary material for: Rates of evolution in stress-related genes are associated with habitat preference in two Cardamine lineages
Source: BMC Evol Biol. 2012 Jan 18;12:7. doi: 10.1186/1471-2148-12-7 (PMC3398273; doi:10.1186/1471-2148-12-7)
Supplement: Additional file 10 — Optimal codons in Cardamine. Putative optimal codons identified in C. resedifolia and C. impatiens. [file 1471-2148-12-7-S10.DOC]

## Additional File 10

**Putative optimal codons in *Cardamine.***

| **Aminoacid code** | **Codon** | ***A. thaliana* genome-wide ^a^** | ***A. thaliana* ^b^** | ***C. resedifolia* ^b^** | ***C. impatiens* ^b^** |
| --- | --- | --- | --- | --- | --- |
| Phe | TTT | 0.41 | 0.67 | 0.78 | 0.72 |
|  | TTC | **2.06** | **1.20** | **1.17** | **1.20** |
| Leu | TTA | 0.25 | 0.15 | 0.30 | 0.42 |
|  | TTG | 1.06 | 1.16 | 1.10 | 1.19 |
|  | CTT | 1.04 | 0.97 | 0.90 | 0.81 |
|  | CTC | **2.59** | **1.48** | **1.52** | **2.07** |
|  | CTA | 0.64 | 0.44 | 0.62 | 0.50 |
|  | CTG | 0.47 | 0.89 | 0.90 | 0.64 |
| Ile | ATT | 0.8 | 1.02 | 0.94 | 1.03 |
|  | ATC | **2.26** | **1.28** | **1.29** | 1.18 |
|  | ATA | 0.21 | 0.13 | 0.30 | 0.28 |
| Val | GTT | 0.86 | 0.99 | 0.92 | 1.06 |
|  | GTC | **2.54** | **1.30** | **1.53** | **1.38** |
|  | GTA | 0.29 | 0.69 | 0.53 | 0.55 |
|  | GTG | 0.9 | 0.92 | 0.91 | 0.81 |
| Ser | TCT | 0.93 | 0.91 | 0.82 | 0.93 |
|  | TCC | **2.52** | 1.21 | 1.10 | 1.00 |
|  | TCA | 0.73 | 0.66 | 1.03 | 0.88 |
|  | TCG | 0.72 | 0.81 | 0.70 | 0.69 |
|  | AGT | 0.55 | 1.21 | 0.98 | 1.22 |
|  | AGC | **1.37** | **1.66** | **1.64** | **1.51** |
| Pro | CCT | 0.8 | 1.06 | 1.04 | 1.10 |
|  | CCC | **2.32** | 1.11 | 1.02 | 0.86 |
|  | CCA | 1.1 | 0.95 | 0.96 | 1.00 |
|  | CCG | 0.6 | 0.87 | 0.96 | 0.82 |
| Thr | ACT | 0.9 | 0.99 | 0.89 | 0.95 |
|  | ACC | **2.86** | 1.25 | **1.67** | **1.44** |
|  | ACA | 0.55 | 0.70 | 0.63 | 0.70 |
|  | ACG | 0.49 | 0.97 | 0.93 | 0.81 |
| Ala | GCT | 1.05 | **1.26** | **1.20** | **1.26** |
|  | GCC | **2.32** | 1.24 | 1.23 | 1.23 |
|  | GCA | 0.5 | 0.54 | 0.67 | 0.61 |
|  | GCG | 0.64 | 0.56 | 0.53 | 0.43 |
| Tyr | TAT | 0.21 | 0.48 | 0.55 | 0.56 |
|  | TAC | **2.41** | **1.26** | **1.33** | **1.29** |
| His | CAT | 0.331 | 1.06 | 0.87 | 0.98 |
|  | CAC | **3.1** | 0.96 | 1.13 | 1.02 |
| Gln | CAA | 0.692 | 0.71 | 0.75 | 0.68 |
|  | CAG | **1.41** | **1.27** | **1.23** | **1.32** |
| Asn | AAT | 0.31 | 0.59 | 0.55 | 0.62 |
|  | AAC | **2.02** | **1.26** | **1.35** | **1.28** |
| Lys | AAA | 0.42 | 0.51 | 0.52 | 0.57 |
|  | AAG | **1.6** | **1.28** | **1.30** | **1.27** |
| Asp | GAT | 0.637 | 0.94 | 0.99 | 1.00 |
|  | GAC | **1.97** | 1.09 | 1.01 | 1.00 |
| Glu | GAA | 0.64 | 0.63 | 0.77 | 0.68 |
|  | GAG | **1.4** | **1.30** | **1.16** | **1.25** |
| Cys | TGT | 0.25 | 0.70 | 0.61 | 0.67 |
|  | TGC | **1.83** | **1.44** | **1.43** | **1.37** |
| Arg | CGT | **2.22** | **1.48** | **1.70** | **1.70** |
|  | CGC | 1.67 | 0.89 | 0.98 | 0.98 |
|  | CGA | 0.41 | 0.72 | 0.46 | 0.49 |
|  | CGG | 0.29 | 0.39 | 0.32 | 0.33 |
|  | AGA | 0.7 | 0.68 | 0.56 | 0.59 |
|  | AGG | **1.18** | 1.15 | **1.50** | **1.29** |
| Gly | GGT | **1.15** | **1.29** | **1.28** | **1.30** |
|  | GGC | **1.49** | 1.26 | **1.53** | 1.06 |
|  | GGA | 0.979 | 0.88 | 0.85 | 0.89 |
|  | GGG | 0.361 | 0.40 | 0.42 | 0.42 |

^a^ Genome-wide relative codon frequencies in *A. thaliana* genes. Frequencies are taken from Chiapello *et al*. (1998) and were calculated as the ratio between codon frequency in high biased and codon frequency in low biased genes. Values in bold identify codons with a significantly higher frequency in high-biased genes than in low-biased genes identified by a multivariate analysis.

^b^ Relative codon frequencies in the ribosomal genes present in our orthologous gene dataset, calculated for *A*. *thaliana*, *C. resedifolia* and *C. impatiens*. Values in bold represent a signiﬁcant increase in the relative frequency of the codon in high biased genes as evaluated by correspondence analysis.
