# Supplementary material for: Rates of evolution in stress-related genes are associated with habitat preference in two Cardamine lineages
Source: BMC Evol Biol. 2012 Jan 18;12:7. doi: 10.1186/1471-2148-12-7 (PMC3398273; doi:10.1186/1471-2148-12-7)
Supplement: Additional file 11 — Codon usage bias in Cardamine genes. Mean codon usage bias, measured as Fop, in the four functional classes considered in this study. Statistical results of the comparisons between functional classes and species are also reported. [file 1471-2148-12-7-S11.DOC]

## Additional File 11

**Codon usage bias, measured as *Fop*, in *Cardamine* genes.**

|  |  |  | ***Fop*** | | |  | ***P* ^d^, in FC *vs*. not in FC** | | | | | | | | | | | | | |
| --- | --- | --- | --- | --- | --- | --- | --- | --- | --- | --- | --- | --- | --- | --- | --- | --- | --- | --- | --- | --- |
|  |  |  | ***C. impatiens* (*Ci*)** |  | ***C. resedifolia* (*Cr*)** |  | ***Fop*** | |  | ***Fop*_avX_ ^e^** | |  | ***Fop*_maxX_ ^e^** | |  | ***Fop*_GC3_ ^e^** | |  | ***Fop*_L_ ^e^** | |
| **Gene FC ^a^** |  | ***n* ^b^** | **mean (SE) ^c^** |  | **mean (SE) ^c^** |  | ***Ci*** | ***Cr*** |  | ***Ci*** | ***Cr*** |  | ***Ci*** | ***Cr*** |  | ***Ci*** | ***Cr*** |  | ***Ci*** | ***Cr*** |
| Cold response (CRG) | In FC | 55 | 0.352 (0.008) |  | 0.317 (0.009) |  | 0.2276 | 0.8151 |  | 0.1169 | 0.6613 |  | 0.1049 | 0.0075 |  | 0.4105 | 0.3702 |  | 0.2500 | 0.8266 |
|  | Not in FC | 2858 | 0.344 (0.001) |  | 0.318 (0.001) |  |  |  |  |  |  |  |  |  |  |  |  |  |  |  |
| Cold  (CGO) | In FC | 56 | 0.374 (0.011) |  | 0.343 (0.011) |  | 0.0036 | 0.0098 |  | 0.7362 | 0.7832 |  | 0.9025 | 0.4280 |  | 0.0027 | 0.0362 |  | 0.0104 | 0.0250 |
|  | Not in FC | 2857 | 0.344 (0.001) |  | 0.318 (0.001) |  |  |  |  |  |  |  |  |  |  |  |  |  |  |  |
| Photosynthesis (PGO) | In FC | 67 | 0.360 (0.009) |  | 0.327 (0.008) |  | 0.0924 | 0.2862 |  | 0.1032 | 0.0053 |  | 0.0885 | 0.0043 |  | 0.1674 | 0.4954 |  | 0.3150 | 0.7705 |
|  | Not in FC | 2846 | 0.344 (0.001) |  | 0.318 (0.001) |  |  |  |  |  |  |  |  |  |  |  |  |  |  |  |
| Stress  (SGO) | In FC | 332 | 0.369 (0.004) |  | 0.341 (0.004) |  | 2×10^-11^ | 7×10^-11^ |  | 0.0521 | 0.1091 |  | 0.0694 | 0.2470 |  | 1×10^-7^ | 1×10^-5^ |  | 5×10^-10^ | 4×10^-9^ |
|  | Not in FC | 2581 | 0.341 (0.001) |  | 0.315 (0.001) |  |  |  |  |  |  |  |  |  |  |  |  |  |  |  |

^a^ Gene functional class.

^b^ Number of genes.

^c^ Mean (standard error).

^d^ Wilcoxon test.

^e^ The comparison was done using the residuals of the correlation between *Fop* and average or maximum expression of the *A. thaliana* orthologue (*Fop*_avX_ and *Fop*_maxX_), GC content at the third codon position (*Fop*_GC3_) and length of the *A. thaliana* orthologue (*Fop*_L_).
